# Correction: Leveraging correlations between variants in polygenic risk scores to detect heterogeneity in GWAS cohorts

**DOI:** 10.1371/journal.pgen.1009158

**Published:** 2020-10-23

**Authors:** Jie Yuan, Hengrui Xing, Alexandre Louis Lamy, The Schizophrenia Working Group of the Psychiatric Genomics Consortium, Todd Lencz, Itsik Pe’er

The second author’s name is spelled incorrectly. The correct name is: Hengrui Xing.
