# Supplementary material for: Leveraging correlations between variants in polygenic risk scores to detect heterogeneity in GWAS cohorts
Source: PLoS Genet. 2020 Sep 21;16(9):e1009015. doi: 10.1371/journal.pgen.1009015 (PMC7529195; doi:10.1371/journal.pgen.1009015)
Supplement: S1 Text — (PDF) [file pgen.1009015.s001.pdf]

Supplementary Methods and Analyses

Negative correlations: an intuitive explanation

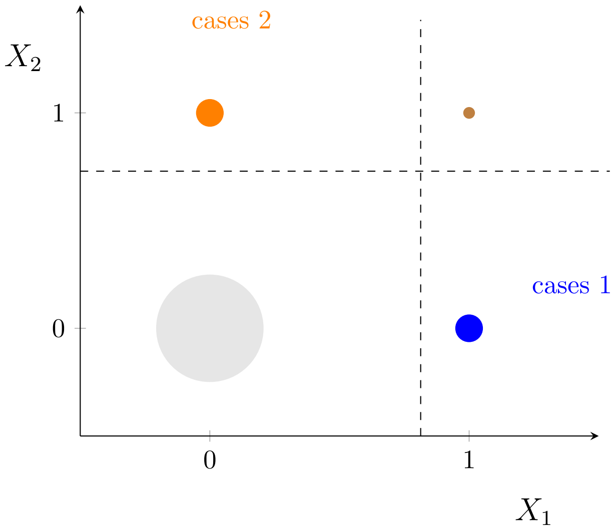

Figure S1-A: **Correlations between PRS predictors are apparent when considering a highly simplified PRS.** Assume a threshold model comprising only two independent haploid SNPs X_1 and X_2, and $\beta_1 = \beta_2 = 1$; i.e. an individual with a single risk-allele for either SNP is classified as a case. The probability of having $X_1 = 1$ given an individual is a case decreases if it is known that $X_2 = 1$. This results in a negative correlation between the two variables.

We demonstrate that among cases that are selected based on a thresholded linear score, as in logistic or liability threshold GWAS models, the correlations between SNPs is expected to be nonzero if those SNPs contribute a nonzero effect to determining the GWAS phenotype. Intuitively, this can be observed in an extreme scenario in which cases and controls are determined by two variables, X_1 and X_2, with a variance explained of 1. This scenario is visualized in S1A. We would like to evaluate the probability of an individual possessing $X_1 = 1$ given that that individual is a case. In the absence of any knowledge of X_2, there approximately an equal chance that the individual is a case because $X_1 = 1$ or $X_2 = 1$, with a typically negligible probability that both are 1.

$$P(\text{case } 1|\text{case } 1 \cup \text{case } 2) =$$ 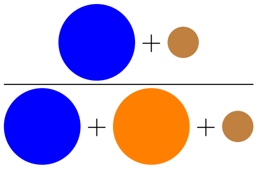 $$\approx 0.5 \qquad (1)$$

However, if it is known that a case has $X_2 = 1$, the case must inhabit the region above the threshold line on the X_2 axis, and so $X_1 = 1$ can only be satisfied if the individual belongs to one of the rare cases with both variables equal to 1.

$$P(\text{case } 1|\text{case } 2) =$$ 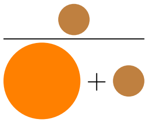 $$\ll 0.5 \qquad (2)$$

Therefore in this extreme scenario X_1 and X_2 are negatively correlated. Had one of the variables decreased the risk score of the individual instead, then by the same logic X_1 and X_2 would be positively correlated. A more complete proof is presented in the following section.

Associated SNPs are correlated among cases in logistic and liability threshold models

Assume there exists a logistic model for disease risk with effects (log odds ratios) for associated SNPs $\{\beta_1, ..., \beta_M\}$. Further, assume we have collected a sample of N cases of a case/control study for which the model predicts disease risk, represented by genotype matrix $X \in \{0, 1, 2\}^{N \times M}$ and labels $Y = \{0, 1\}^N$. All genotypes in X are independently sampled in controls and are thus uncorrelated. We demonstrate that among cases, the correlation r between any two SNPs in X with nonzero effect is nonzero. Specifically, given two SNPs j and k with effects β_j and β_k,

1. $\beta_j = 0 \cup \beta_k = 0 \implies r_{jk} = 0$
2. $\text{sign}(\beta_j) = \text{sign}(\beta_k) \implies r_{jk} < 0$
3. $\text{sign}(\beta_j) \neq \text{sign}(\beta_k) \implies r_{jk} > 0$

The correlation between SNPs j and k in the sample, represented as X_j and X_k, is by definition

$$r_{jk} = \frac{\mathbb{E}[X_j X_k] - \mathbb{E}[X_j]\mathbb{E}[X_k]}{\sqrt{\mathbb{E}[X_j^2] - \mathbb{E}[X_j]^2}\sqrt{\mathbb{E}[X_k^2] - \mathbb{E}[X_k]^2}} \tag{3}$$

and so the sign of r_{jk} is determined by the sign of the numerator. Here $E[X_j]$ represents the expected risk-allele count of SNP j within the set of cases.

Using the law of total expectation, rewrite the expectation of the product of X_j and X_k as an expectation over X_j conditional on X_k:

$$\begin{aligned} \mathbb{E}[X_j X_k] =& \mathbb{E}_{X_k}[\mathbb{E}[X_j X_k | X_k]] \\ =& \mathbb{E}[X_k \mathbb{E}[X_j | X_k]] \end{aligned} \tag{4}$$

This term is substituted into the numerator of the correlation in equation 3 and expanded over the marginalization of $X_k = \{0, 1, 2\}$, with the $X_k = 0$ term canceling.

$$\begin{aligned} \mathbb{E}[X_j X_k] - \mathbb{E}[X_j]\mathbb{E}[X_k] =& \mathbb{E}[X_k \mathbb{E}[X_j | X_k]] - \mathbb{E}[X_k \mathbb{E}[X_j]] \\ =& \Big[p(X_k = 1)\mathbb{E}[X_j | X_k = 1] + 2p(X_k = 2)\mathbb{E}[X_j | X_k = 2] \Big] - \\ & \Big[p(X_k = 1)\mathbb{E}[X_j] + 2p(X_j = 2)\mathbb{E}[X_j] \Big] \end{aligned} \tag{5}$$

This expression determines the sign of the correlation between SNPs X_j and X_k. The expectation $E[X_j]$ can be expressed as a sum of conditional expectations on X_j. After rearranging terms:

$$
\begin{aligned}
\mathbb{E}[X_jX_k] - \mathbb{E}[X_j]\mathbb{E}[X_k] =& p(X_k = 1)\left[\mathbb{E}[X_j|X_k = 1] - \left[\begin{array}{r}\mathbb{E}[X_j|X_k = 0]p(X_k = 0)+\\ \mathbb{E}[X_j|X_k = 1]p(X_k = 1)+\\ \mathbb{E}[X_j|X_k = 2]p(X_k = 2)\end{array}\right]\right]+\\
& 2p(X_k = 2)\left[\mathbb{E}[X_j|X_k = 2] - \left[\begin{array}{r}\mathbb{E}[X_j|X_k = 0]p(X_k = 0)+\\ \mathbb{E}[X_j|X_k = 1]p(X_k = 1)+\\ \mathbb{E}[X_j|X_k = 2]p(X_k = 2)\end{array}\right]\right]\\
=& p(X_k = 1)\left[\mathbb{E}[X_j|X_k = 1](1 - p(X_k = 1)) - \left[\begin{array}{r}\mathbb{E}[X_j|X_k = 0]p(X_k = 0)+\\ \mathbb{E}[X_j|X_k = 2]p(X_k = 2)\end{array}\right]\right]+\\
& 2p(X_k = 2)\left[\mathbb{E}[X_j|X_k = 2](1 - p(X_k = 2)) - \left[\begin{array}{r}\mathbb{E}[X_j|X_k = 0]p(X_k = 0)+\\ \mathbb{E}[X_j|X_k = 1]p(X_k = 1)\end{array}\right]\right]\\
=& p(X_k = 1)\left[\mathbb{E}[X_j|X_k = 1](\begin{array}{r}p(X_k = 0)+\\ p(X_k = 2\end{array})) - \left[\begin{array}{r}\mathbb{E}[X_j|X_k = 0]p(X_k = 0)+\\ \mathbb{E}[X_j|X_k = 2]p(X_k = 2)\end{array}\right]\right]+\\
& 2p(X_k = 2)\left[\mathbb{E}[X_j|X_k = 2](\begin{array}{r}p(X_k = 0)+\\ p(X_k = 1)\end{array}) - \left[\begin{array}{r}\mathbb{E}[X_j|X_k = 0]p(X_k = 0)+\\ \mathbb{E}[X_j|X_k = 1]p(X_k = 1)\end{array}\right]\right]
\end{aligned} \tag{6}
$$

After collecting the conditional expectation terms:

$$
\begin{aligned}
\mathbb{E}[X_jX_k] - \mathbb{E}[X_j]\mathbb{E}[X_k] =& \mathbb{E}[X_j|X_k = 0]\big(- p(X_k = 0)p(X_k = 1) - 2p(X_k = 2)p(X_k = 0)\big)+\\
& \mathbb{E}[X_j|X_k = 1]\left(\begin{array}{r}p(X_k = 1)p(X_k = 0) + p(X_k = 1)p(X_k = 2)-\\ 2p(X_k = 2)p(X_k = 1)\end{array}\right)+\\
& \mathbb{E}[X_j|X_k = 2]\left(\begin{array}{r}-p(X_k = 1)p(X_k = 2) + 2p(X_k = 2)p(X_k = 0)+\\ 2p(X_k = 2)p(X_k = 1)\end{array}\right)
\end{aligned} \tag{7}
$$

Assuming Hardy-Weinberg equilibrium in cases, probabilities of X_k can be expressed as a function of the risk-allele frequency p_k.

$$
p(X_k = 0) = (1 - p_k)^2 \qquad p(X_k = 1) = 2p_k(1 - p_k) \qquad p(X_k = 2) = p_k^2 \tag{8}
$$

After substitution of these expressions into equation 7, the result is a sum of polynomial functions of p_k.

$$
\begin{aligned}
\mathbb{E}[X_jX_k] - \mathbb{E}[X_j]\mathbb{E}[X_k] =& \mathbb{E}[X_j|X_k=0]\big(-2p_k^3+4p_k^2-2p_k\big)+ \\
& \mathbb{E}[X_j|X_k=1]\big(4p_k^3-6p_k^2+2p_k\big)+ \\
& \mathbb{E}[X_j|X_k=2]\big(-2p_k^3+2p_k^2\big)
\end{aligned} \tag{9}
$$

Note that when $\mathbb{E}[X_j|X_k=0]=\mathbb{E}[X_j|X_k=1]=\mathbb{E}[X_j|X_k=2]$, equation 9 reduces to 0 for all values of p_k. When $\mathbb{E}[X_j|X_k=0]<\mathbb{E}[X_j|X_k=1]<\mathbb{E}[X_j|X_k=2]$, equation 9 is strictly positive over the $[0,1]$ domain of p_k whereas when $\mathbb{E}[X_j|X_k=0]>\mathbb{E}[X_j|X_k=1]>\mathbb{E}[X_j|X_k=2]$, the function is strictly negative over the domain. Next we must prove that the former inequality would result if the signs of the effect sizes of X_j and X_k were different, whereas the latter inequality would result if the signs were the same.

For any value of X_k, the conditional expectation of X_j in cases can be expanded according to Bayes' rule:

$$
\begin{aligned}
\mathbb{E}[X_j|X_k] &= \sum_{X_j\in\{0,1,2\}} X_j p(X_j|X_k, Y=1) \\
&= \sum_{X_j\in\{0,1,2\}} X_j \frac{p(Y=1|X_j,X_k)p(X_j)}{\sum_{X_j\in\{0,1,2\}} p(Y=1|X_j,X_k)p(X_j)} \\
&= \frac{\sum_{X_j\in\{0,1,2\}} X_j p(Y=1|X_j,X_k)p(X_j)}{\sum_{X_j\in\{0,1,2\}} p(Y=1|X_j,X_k)p(X_j)} \\
&= \frac{\begin{array}{c} p(Y=1|X_j=1,X_k)p(X_j=1)+ \\ 2p(Y=1|X_j=2,X_k)p(X_j=2) \end{array}}{\begin{array}{c} p(Y=1|X_j=0,X_k)p(X_j=0)+ \\ p(Y=1|X_j=1,X_k)p(X_j=1)+ \\ p(Y=1|X_j=2,X_k)p(X_j=2)) \end{array}}
\end{aligned} \tag{10}
$$

The probability $p(Y=1|X_j,X_k)$ is the probability of an individual being a case and is determined by a logistic function with fixed values for X_j and X_k. The numerator of Equation 10 differs from the denominator by replacing $p(Y=1|X_j=0,X_k)p(X_j=0)$ with a second $p(Y=1|X_j=2,X_k)p(X_j=2)$ term. Consider the expression $\frac{p(Y=1|X_j=2,X_k)p(X_j=2)}{p(Y=1|X_j=0,X_k)p(X_j=0)}$. We would like to show first that the magnitude of

this term decreases as $X_k \in \{0,1,2\}$ increases. Express the probabilities as logistic functions

$$\frac{\frac{1}{1+\exp\left(-(2b_j+X_kb_k+\mathbb{E}[Xb]_{-jk})\right)}}{\frac{1}{1+\exp\left(-(X_kb_k+\mathbb{E}[Xb]_{-jk})\right)}}\frac{p(X_j=2)}{p(X_k=0)} = \frac{1+\exp\left(-(X_kb_k+\mathbb{E}[Xb]_{-jk})\right)}{1+\exp\left(-(2b_j+X_kb_k+\mathbb{E}[Xb]_{-jk})\right)}\frac{p(X_j=2)}{p(X_k=0)} \tag{11}$$

where b_j and b_k are the odds ratios of SNPs j and k, respectively, and $\mathbb{E}[Xb]_{-jk}$ is the expected contribution of the remaining undetermined SNPs when SNPs j and k are fixed. If the effect size of X_k is positive, the contribution of a particular value of X_k can be represented as adding a constant term c within a separate exponential term.

$$\begin{aligned}
\frac{1+\exp(-(\mathbb{E}[Xb]_{-jk}+c))}{1+\exp(-(2b_j+\mathbb{E}[Xb]_{-jk}+c))}\frac{p(X_j=2)}{p(X_k=0)} &= \frac{1+\exp(-(a+c))}{1+\exp(-(b+a+c))}D \\
&= \frac{1+\exp(-a)\exp(-c)}{1+\exp(-(b+a))\exp(-c)}D \\
&= \frac{\big[1+\exp(-a)\big]\exp(-c)+1-\exp(-c)}{\big[1+\exp(-(b+a))\big]\exp(-c)+1-\exp(-c)}D \\
&= \frac{\big[1+\exp(-a)\big]+\exp(c)-1}{\big[1+\exp(-(b+a))\big]+\exp(c)-1}D \\
&= \frac{\big(1+\exp(-\mathbb{E}[Xb]_{-jk})\big)+\exp(c)-1}{\big(1+\exp(-(2b_j+\mathbb{E}[Xb]_{-jk}))\big)+\exp(c)-1}\frac{p(X_j=2)}{p(X_k=0)}
\end{aligned} \tag{12}$$

Therefore, fixing X_k to a particular value has the effect of adding a $\exp(c)-1$ term to the numerator and denominator of the ratio. Additionally, as the number of risk-alleles of X_k is increased, the value of c and thus $\exp(c)-1$ increases. This allows us to take advantage of the following Lemma:

Lemma: for strictly positive values of c, d, e with $c > d$, $\frac{c}{d} > \frac{c+e}{d+e}$. $c > d$ is satisfied for all conditional expectations of X_j provided that the effect of X_j is positive.

$$
\begin{aligned}
c >& d \\
ce + cd >& de + cd \\
c(e+d) >& d(e+c) \\
\frac{c}{d} >& \frac{c+e}{d+e}
\end{aligned} \tag{13}
$$

Now substitute the fraction $\frac{p(Y=1|X_j=2,X_k)p(X_j=2)}{p(Y=1|X_j=0,X_k)p(X_j=0)}$ for $\frac{c}{d}$. When the value of X_k is fixed, the change to the probability is equivalent to adding a constant e to both the numerator and denominator, as shown in Equation 12. By the above lemma, an increase in X_k and thus an increase in the magnitude of e reduces the value of $\frac{p(Y=1|X_j=2,X_k)p(X_j=2)}{p(Y=1|X_j=0,X_k)p(X_j=0)}$ as $\frac{c}{d}$.

Next, rewrite the fraction as $p(Y = 1|X_j = 2, X_k)p(X_j = 2) = ap(Y = 1|X_j = 0, X_k)p(X_j = 0)$ where a is some positive constant greater than 1, as with all other variables fixed and a positive effect b_j, $X_j = 2$ will always increase the probability of generating a case vs $X_j = 0$. We can then eliminate $p(Y = 1|X_j = 2, X_k)p(X_j = 2)$ in Equation 10. For simplicity we have also made the substitution $p_x = p(Y = 1|X_j = x, X_k)p(X_j = x)$:

$$
\begin{aligned}
\mathbb{E}[X_j|X_k] =& \frac{\begin{array}{c} p(Y=1|X_j=1,X_k)p(X_j=1)+ \\ 2p(Y=1|X_j=2,X_k)p(X_j=2) \end{array}}{\begin{array}{c} p(Y=1|X_j=0,X_k)p(X_j=0)+ \\ p(Y=1|X_j=1,X_k)p(X_j=1)+ \\ p(Y=1|X_j=2,X_k)p(X_j=2)) \end{array}} \\
=& \frac{p_1 + 2p_2}{p_0 + p_1 + p_2} \\
=& \frac{p_1 + 2ap_0}{p_1 + (1+a)p_0}
\end{aligned} \tag{14}
$$

The first and second derivatives of this function with respect to a are:

$$
\begin{aligned}
\frac{d}{da}\left[\frac{p_1 + 2ap_0}{p_1 + (1+a)p_0}\right] &= \frac{p_0(2p_0 + p_1)}{(p_0 + p_1 + p_0 a)^2} \\
\frac{d^2}{da^2}\left[\frac{p_1 + 2ap_0}{p_1 + (1+a)p_0}\right] &= \frac{-2p_0^2(2p_0 + p_1)}{(p_0 + p_1 + p_0 a)^3}
\end{aligned} \tag{15}
$$

For positive values of a, the first derivative is always positive, and the second derivative is always negative, indicating that this is a monotonic, concave function. We have previously established that as X_k increases in magnitude, a decreases; that is, $a_{k=0} > a_{k=1} > a_{k=2}$. As $E[X_j|X_k]$ is a monotonically increasing function of a, then the previous inequality is likewide true for $E[X_j|X_k]$. Therefore, $\mathbb{E}[X_j|X_k = 0, Y = 1] > \mathbb{E}[X_j|X_k = 1, Y = 1] > \mathbb{E}[X_j|X_k = 2, Y = 1]$. Conversely, when X_j has an effect size less than 0, and thus opposite that of X_j, then $\mathbb{E}[X_j|X_k = 0, Y = 1] < \mathbb{E}[X_j|X_k = 1, Y = 1] < \mathbb{E}[X_j|X_k = 2, Y = 1]$.

Prediction of heterogeneity scores in homogeneous (null) cohorts

The heterogeneity score relies on the weighted difference in correlations between cases and controls. The test for heterogeneity assumes in the null situation that a cohort of cases is completely homogeneous, i.e. sampled and thresholded using the same polygenic risk score model. Therefore expected sample correlations between every pair of predictors X_i and X_j (either SNP allele counts or gene expression measurements) are computed assuming that all individuals are identically sampled cases.

$$
r(X_i, X_j) = \frac{\mathbb{E}[X_i X_j] - \mathbb{E}[X_i]\mathbb{E}[X_j]}{\sqrt{\mathbb{E}[X_i^2] - \mathbb{E}[X_i]^2}\sqrt{\mathbb{E}[X_j^2] - \mathbb{E}[X_j]^2}} \tag{16}
$$

By Bayes theorem, each of these expectations can be computed from the posterior probabilities of the predictor values given an individual is a case ($y = 1$):

$$
\begin{aligned}
\mathbb{E}[X_i|\text{y=1}] =& \frac{\int X_i P(\text{y=1}|X_i) P(X_i) dX_i}{\int P(\text{y=1}|X_i = z) P(X_i = z) dz} \\
\mathbb{E}[X_i^2|\text{y=1}] =& \frac{\int X_i^2 P(\text{y=1}|X_i) P(X_i) dX_i}{\int P(\text{y=1}|X_i = z) P(X_i = z) dz} \\
\mathbb{E}[X_i X_j|\text{y=1}] =& \frac{\int \int X_i X_j P(\text{y=1}|X_i, X_j) P(X_i, X_j) dX_i dX_j}{\int \int P(\text{y=1}|X_i = z, X_j = w) P(X_i = z, X_j = w) dz dw}
\end{aligned}
\tag{17}
$$

The prior probability for any predictor $P(X_i)$ when X are SNPs in Hardy-Weinberg equilibrium is simply the binomial distribution parameterized by the risk-allele frequency in controls. As we assume that in controls SNPs are sampled from independent loci, their joint distributions are simply the product of these priors.

However, we would like to calculate the expected null score of a homogeneous set of cases when the input predictors are both quantitative and correlated. In particular, we consider the scenario of scoring heterogeneity in transcriptome-wide gene associations as described in Mancuso et al. [1]. Assume, over N individuals, G is a genotype matrix comprising S SNPs and X is a gene expression matrix comprising M genes. Genes are determined by linear models over SNPs with SNP-gene effect sizes $\beta_{(S \times M)}$.

$$
\begin{aligned}
X_i \sim N(\beta_i G, 1 - V_{G_i}) \quad \forall i \in 1, ..., M \\
V_{G_i} = 2 \sum_{s=1}^{S} \beta_{is}^2 p_s (1 - p_s)
\end{aligned}
\tag{18}
$$

where V_{G_i} is the variance explained of the SNPs G on gene X_i and p_s is the allele frequency of SNP s. Case/control disease status y is then determined by a liability threshold model over genes with liability effect sizes $\alpha_{(M \times 1)}$.

$$P(y=1|X) = \Phi\left(\frac{X\alpha - T}{\sqrt{1-V_E}}\right), \quad V_E = \sum_{i=1}^{M} \alpha_i^2$$
$$P(y=1|X_i) = \Phi\left(\frac{X_i\alpha_i - T}{\sqrt{1-\alpha_i^2}}\right) \tag{19}$$

When the genes X are functions of mutually exclusive sets of SNPs, then equation 19 can be plugged into the case expectation terms in equation 17 and solved using numeric integration. These expectations are then used to solve the expected correlation $r(X_i, X_j)$ in homogeneous cases.

When multiple genes in X are associated with a common subset of SNPs, then the expected correlation between these genes is nonzero. Therefore when a particular X_i is fixed to an observed value, the expected values of the remaining X_{-i} are also altered, which in turn modify the value of $P(y=1|X_i)$. To calculate the correct expected correlation, we make use of the following lemma:

Lemma: Given a k-dimensional multivariate normal distribution $Z \sim N(\mu, \Lambda)$, assume some subset of components $b \subset k$ are fixed with values X_b. Define $\mu_{\cdot}$ and $\Lambda_{\cdot\cdot}$ as the partitions of the mean and covariance of a multivariate normal distribution corresponding to the specified component set. Then the remaining components $a = k \setminus b$ are multivariate-normal distributed with the following mean and covariance [2]

$$\mu_{a|b} = \mu_a + \Lambda_{ab}\Lambda_{bb}^{-1}(X_b - \mu_b) \tag{20}$$
$$\Lambda_{a|b} = \Lambda_{aa} - \Lambda_{ab}\Lambda_{bb}^{-1}\Lambda_{ba} \tag{21}$$

As the genes are defined as linear functions of SNPs, the covariance between any two genes is

$$\mathrm{Cov}(X_i, X_j) = \Lambda_{ij} = \mathrm{Cov}(\beta_i G, \beta_j G) = \sum_{s=1}^{S} \beta_{is}\beta_{js} V_{G_i} \tag{22}$$

Calculation of the expectation terms in equation 17 requires fixing $X_b = X_i$ or $X_b = \langle X_i, X_j \rangle$ to some value: the indices of these variables serve as the subset b while those of the rest of the M genes serve as subset $a = \{1, ..., M\} \setminus b$. As the genes in a are correlated with those in b, fixing values for b further informs the probabilities of a and thus the mean and variance of the liability distribution over genes. The case probability given values for subset b shown in equation 19 now becomes

$$P(y = 1|X_b) = \Phi\left(\frac{X_b\alpha_b + (\Lambda_{ab}\Lambda_{bb}^{-1}X_b)\alpha_a - T}{\sqrt{1 - \sum_{i\in b}\alpha_i^2 + \sum_{w\in\{1,...,M\}\setminus b}\left[\alpha_w^2 + 2\sum_{z\in\{w+1,...,M\}\setminus b}\alpha_w\alpha_z\Lambda_{wz}\right]}}\right) \tag{23}$$

This expression now accounts for correlations between genes arising from shared SNP effects and can be substituted for equation 19 when calculated the expected correlation in homogeneous cases.

Normality of polygenic risk scores

The central limit theorem applies when the random variables to be summed are independent and identically distributed. When creating polygenic risk scores, independence can be ensured to a degree by carefully selecting SNPs for inclusion which are not in LD with one another. Any set of SNPs, however, will not be identically distributed due to differing allele frequencies and scaling by effect sizes. Additionally, while the prior distribution of effect sizes is itself assumed to be normal, we cannot assume that the set of significant effect sizes comprising the PRS is likewise normally distributed. But, we can still show that polygenic risk scores given these specifications should converge to a normal distribution using Lyapunov's theorem:

Given $\{X_1, ..., X_M\}$ independent random variables and variance of the sum $s^2 = \Sigma_{j=1}^{M}\sigma_j^2$, for any

$\delta > 0$, if

$$\lim_{M\to\infty} \frac{1}{s^{2+\delta}} \sum_{j=1}^{M} \mathbb{E}\Big[|X_j - \mu_j|^{2+\delta}\Big] = 0 \tag{24}$$

then

$$\frac{1}{s}\sum_{j=1}^{M} X_j - \mu_j \to N(0,1) \tag{25}$$

First, we assume that SNPs are Bernoulli distributed variables scaled by effect sizes β_j. If desired, diploidy can be represented by sampling from two Bernoulli variables for each SNP. Secondly, we assume that the magnitudes of the effect sizes β_j are larger than some value ϵ, a reasonable assumption if these are SNPs discovered through GWAS. This prevents scenarios such as $\beta_1 > 0$ and $\beta_2, ..., \beta_M = 0$, for which the limit does not converge. Given these assumptions, each SNP j in the summed PRS contributes a variance of $\beta_j^2 p_j(1-p_j)$. Plugging into Lyapunov's expression yields the following:

$$\begin{aligned}
\lim_{M\to\infty} \frac{1}{s^3}\sum_{j=1}^{M} \mathbb{E}\Big[|X_j - \mu_j|^3\Big] &= \lim_{M\to\infty} \frac{\sum_{j=1}^{M} p_j|\beta_j^3|(1-p_j)^3 + (1-p_j)|\beta_j^3|p_j^3}{\Big[\sum_{j=1}^{M} \beta_j^2 p_j(1-p_j)\Big]^{\frac{3}{2}}} \\
&= \lim_{M\to\infty} \frac{\sum_{j=1}^{M} |\beta_j^3| p_j(1-p_j)\Big[(1-p_j)^2 + p_j^2\Big]}{\Big[\sum_{j=1}^{M} \beta_j^2 p_j(1-p_j)\Big]^{\frac{3}{2}}} \\
&\leq \lim_{M\to\infty} \frac{\sum_{j=1}^{M} |\beta_j^3| p_j(1-p_j)}{\Big[\sum_{j=1}^{M} \beta_j^2 p_j(1-p_j)\Big]^{\frac{3}{2}}} \overset{|\beta|>\epsilon}{=} 0
\end{aligned} \tag{26}$$

Therefore a sum of scaled Bernoulli variables should converge to a normal distribution even when SNPs are not identically distributed, and effect sizes are not normally distributed.

Validation of sample disease prevalences in logistic and liability threshold models

We demonstrate in panel A of Fig 2 that simulated cases sampled from a logistic model and its conversion into a liability threshold model exhibit very similar acertainment for effect alleles, with slight

deviations occurring as odds ratios increase. Here we confirm that disease prevalences of the simulated cohorts also do not deviate significantly between the two models. Given a set of simulated individuals whose genotypes are randomly sampled, we assign case/control statuses to the same individuals using either a logistic model or a liability threshold model with the same parameters as Fig 2. The fraction of individuals assigned as cases from each model is plotted in S1 Fig. Both models were set to produce a desired prevalence of 0.01, with varying odds ratios specified by color. We observe that the logistic model produces slightly inflated sample prevalences reaching a maximum of 0.0175 when odds ratios are large, versus a desired prevalence of 0.01. We suspect the inflation occurs because logistic regression does not explicitly model the variance of the log-odds. For a log-odds distribution mean-centered at zero, if the odds ratios increase, so does the log-odds variance, allowing a slightly larger fraction of individuals to be sampled as cases. This would also explain the decrease in ascertainment of effect alleles relative to the liability threshold model seen in panel A of Fig 2, as increasing the log-odds variance effectively relaxes the criteria for selecting a case. However, these biases in the logistic model occur only at large odds ratios unrepresentative of the vast majority of polygenic effects. These biases are also unlikely to be the primary source of the discrepancy in correlation patterns between the logistic and liability threshold models in panel B of Fig 2, as the heterogeneity scores of the two models begin to diverge at even small odds ratios.

Additional technical details on the Risch model

The Risch model assumes disease risk is determined by a product of relative risks.

$$P(y=1|X_i) = A \prod_{m=1}^{M} RR_m^{X_{im}} \tag{27}$$

The constant A is set such that the expected fraction of cases in a random sample of genotypes is equal to the desired prevalence. Therefore for a prevalence V we set $A = \frac{V}{\mathbb{E}\left[\prod_{m=1}^{M} RR_m^{X_m}\right]}$. In our simulations, we assume that all M SNPs possess a constant odds ratio OR and allele frequency p, and further that given a small prevalence of 0.01, the odds ratio can be substituted for the relative risk. These simplifications allow us to define q as the total effect allele count across all SNPs, which is approximately normal distributed for large M with mean $\mu = Mp$ and variance $\sigma^2 = Mp(1-p)$. We can then derive an expression for the

expected value in the denominator of A:

$$\begin{aligned}
\mathbb{E}[\prod_{m=1}^{M} RR_m^{X_m}] &\approx \int OR^q \frac{1}{\sqrt{2\pi\sigma^2}} e^{-\frac{(q-\mu)^2}{2\sigma^2}} dq \\
&= \frac{1}{\sqrt{2\pi\sigma^2}} \int e^{(\log OR)q - \frac{(q-\mu)^2}{2\sigma^2}} dq \\
&= \frac{1}{\sqrt{2\pi\sigma^2}} \int e^{\frac{1}{2\sigma^2}[2\sigma^2(\log OR)q - q^2 + 2q\mu - \mu^2]} dq \\
&= \frac{1}{\sqrt{2\pi\sigma^2}} \int e^{-\mu^2 + (\sigma^2(\log OR)+\mu)^2} e^{-\frac{(q-(\sigma^2(\log OR)+\mu))}{2\sigma^2}} dq \\
&= e^{-\mu^2 + (\sigma^2(\log OR)+\mu)^2} \frac{1}{\sqrt{2\pi\sigma^2}} \int e^{-\frac{(q-(\sigma^2(\log OR)+\mu))}{2\sigma^2}} dq \\
&= e^{-\mu^2 + (\sigma^2(\log OR)+\mu)^2}
\end{aligned} \tag{28}$$

Therefore in equation 1 of the Methods specifying the Risch model, we set the constant $A = \frac{V}{\exp(-(Mp)^2 + ((Mp(1-p))^2(\log OR) + Mp)^2)}$ where V is the prevalence, and all M SNPs share odds ratio OR and allele frequency p.

While the Risch model is calculated using relative risks, relative risks can be approximated by odds ratios if the prevalence is small [3]. Zhang and Kai [4] propose the following equation to convert from odds ratios to an approximation of relative risk (or risk ratio) if the disease prevalence V is known.

$$RR = \frac{OR}{(1-V) + V * OR} \tag{29}$$

To verify relative risks do not deviate significantly from odds ratios, we plug in the simulation parameters used in panel A of S2 Fig, with $V = 0.01$ and $OR = 1.2$, to obtain a relative risk of $RR = 1.198$, and with $V = 0.01$ and $OR = 1.06$ in panel B of S2 Fig, to obtain $RR = 1.059$. As the odds ratios closely approximate their corresponding relative risks, we substituted the odds ratios directly into the Risch model for simulations in S2 Fig.

Additional details on derivation of CLiP-X weight functions

Starting with the expression for correlation weighted by π,

$$r_{jk} = \frac{\begin{array}{c}\pi\mathbb{E}\big[X_j^- X_k^-\big] + (1-\pi)\mathbb{E}\big[X_j^+ X_k^+\big] - \\ (\pi\mu_j^- + (1-\pi)\mu_j^+)(\pi\mu_k^- + (1-\pi)\mu_k^+)\end{array}}{\sqrt{\begin{array}{l}\pi\mathbb{E}\big[(X_j^-)^2\big] + \\ \quad (1-\pi)\mathbb{E}\big[(X_j^+)^2\big] - \\ \qquad \big[\pi\mu_j^- + (1-\pi)\mu_j^+\big]^2\end{array}}\sqrt{\begin{array}{l}\pi\mathbb{E}\big[(X_k^-)^2\big] + \\ \quad (1-\pi)\mathbb{E}\big[(X_k^+)^2\big] - \\ \qquad \big[\pi\mu_k^- + (1-\pi)\mu_k^+\big]^2\end{array}}} \tag{30}$$

Taking the derivative with respect to π at $\pi = 0$ allows the cancellation of many terms. If r_{jk} is expressed as $r_{jk} = \frac{a}{bc}$, where a is the entire numerator and b and c are the two standard deviation terms in the denominator, then $\frac{\partial}{\partial\pi} r_{jk}\big|_{\pi=0} = \frac{a'bc - a(b'c + bc')}{b^2c^2}$. With $\pi = 0$, the second term in numerator simplifies to $\mathbb{E}[X_j^+ X_k^+] - \mu_j^+ \mu_k^+$, which equals zero when we assume there is no correlation between SNPs in a homogeneous case or control set. Therefore we evaluate $\frac{\partial}{\partial\pi} r_{jk}\big|_{\pi=0} = \frac{a'}{bc}$.

From our assumption that there is no correlation within cases or controls

$$\mathbb{E}[X_j^+ X_k^+] = \mu_j^+ \mu_k^+ \tag{31}$$

$$\mathbb{E}[(X_j^+)^2] = (\mu_j^+)^2 + (\sigma_j^+)^2 \tag{32}$$

where $(\sigma_j^+)^2$ is the variance of SNP j within sub-cohort $+$. Lastly, at $\pi = 0$, each standard deviation term in the denominator reduces to

$$\mathbb{E}[(X_j^-)^2] - (\mu_j^-)^2 = \big[(\mu_j^-)^2 + (\sigma_j^-)^2\big] - (\mu_j^-)^2 = (\sigma_j^-)^2 \tag{33}$$

This yields the heterogeneity test statistic for continuous inputs.

$$w_{jk} = \frac{\partial}{\partial \pi} r_{jk} \bigg|_{\pi=0} \tag{34}$$

$$= \frac{\mu_j^+ \mu_k^+ - \mu_j^+ \mu_k^- - \mu_j^- \mu_k^+ + \mu_j^- \mu_k^-}{\sigma_j^- \sigma_k^-} \tag{35}$$

$$= \frac{(\mu_j^+ - \mu_j^-)(\mu_k^+ - \mu_k^-)}{\sigma_j^- \sigma_k^-} \tag{36}$$

Supplemental References

1. Mancuso N, Shi H, Goddard P, Kichaev G, Gusev A, Pasaniuc B. Integrating gene expression with summary association statistics to identify genes associated with 30 complex traits. The American Journal of Human Genetics. 2017;100(3):473–487.

2. Bishop CM. Pattern recognition and machine learning. springer; 2006.

3. Wang Z, et al. Converting odds ratio to relative risk in cohort studies with partial data information. J Stat Softw. 2013;55(5):1–11.

4. Zhang J, Kai FY. What's the relative risk?: A method of correcting the odds ratio in cohort studies of common outcomes. Jama. 1998;280(19):1690–1691.
