# Supplementary material for: Leveraging correlations between variants in polygenic risk scores to detect heterogeneity in GWAS cohorts
Source: PLoS Genet. 2020 Sep 21;16(9):e1009015. doi: 10.1371/journal.pgen.1009015 (PMC7529195; doi:10.1371/journal.pgen.1009015)
Supplement: S2 Text — We assume the input predictors are normally distributed gene expression transcripts generated from linear functions of sampled binomial SNPs and total SNP variance explained VG2. Then cases and controls are sampled based on a liability threshold model on these gene expression variables. (PDF) [file pgen.1009015.s002.pdf]

```
Function SampleCLiP-X
Input: N, M, L, V_G^2, V_E^2, π  Output: genotypes X_{N×M}, expression Z_{N×L}
T = Φ^{-1}(1 − .01); // threshold from 0.01 prevalence
/* define SNP and expression summary statistics */
p_{i∈[1,M]} = 0.5;  β_{i∈[1,M]} = sqrt( (V_G^2/M) / Var(X_{·i}) );  α_{i∈[1,L]} = sqrt( (V_E^2/L) / (Var(Z_{·i}) + 2 Σ_{j∈L} Cov(Z_{·i}, Z_{·j})) );
/* Generate control genotypes and expression */
for n in [1, (1 − π)N], m in [1, M] do
    X^0_{n,m} ~ Binom(2, p_m);
end
Z^0 ~ Normal(X^0 · β, 1 − V_G^2);
/* Generate case genotypes and expression */
X = []; Z = [];
while nrows(X) = nrows(Z) < πN do
    for m in [1, M] do
        x_m ~ Binom(2, p_m);
    end
    z ~ Normal(x · β, 1 − V_G^2);
    y ~ Normal(z · α, 1 − V_E^2);
    if y ≥ T then
        append(X, x);
        append(Z, z);
    end
end
/* Simulate heterogeneity by concatenating generated cases and controls */
X = concatenate(X, X^0);
Z = concatenate(Z, Z^0);
```

S2 Text. **Sampling procedure for heterogeneous cases with quantitative predictors.** We assume the input predictors are normally distributed gene expression transcripts generated from linear functions of sampled binomial SNPs and total SNP variance explained V_G^2. Then cases and controls are sampled based on a liability threshold model on these gene expression variables.
