# Supplementary material for: Leveraging correlations between variants in polygenic risk scores to detect heterogeneity in GWAS cohorts
Source: PLoS Genet. 2020 Sep 21;16(9):e1009015. doi: 10.1371/journal.pgen.1009015 (PMC7529195; doi:10.1371/journal.pgen.1009015)
Supplement: S3 Text — In lieu of explicit case/control labels by which to generate heterogeneous cohorts, we define ‘cases’ as individuals whose phenotypes are a function of their PRSs, whereas ‘controls’ have phenotypes that are sampled completely randomly from the same distribution. (PDF) [file pgen.1009015.s003.pdf]

**Function** SampleCLiP-Y

**Input:** $N, M, h^2_{SNP}, cohort = \{hom, het\}$

**Output:** $X_{N\times M}, Y_{N\times 1}$

```
/* define SNP and expression summary statistics */
```

$p_{i\in[1,M]} = 0.5; \quad \beta_{i\in[1,M]} = \sqrt{\frac{h^2_{SNP}/M}{\mathrm{Var}(X_{\cdot i})}};$

**for** $n$ in $[1, N]$, $m$ in $M$ **do**

  $X_{nm} \sim \mathrm{Binom}(2, p_m);$

**end**

```
/* if heterogeneous, generate mislabeled individuals */
```

**if** $cohort = het$ **then**

  $Y_{[1,N/2]} \sim \mathrm{Normal}(X_{[1,N/2],\cdot} \cdot \beta, 1 - h^2_{SNP});$

  $Y^0_{[1,N/2]} \sim \mathrm{Normal}(\mathbb{E}[X\beta], 1 - h^2_{SNP});$

  $Y = \mathrm{concatenate}(Y, Y^0)$

**else**

  $Y \sim \mathrm{Normal}(X \cdot \beta, 1 - h^2_{SNP});$

**end**

S3 Text. **Sampling procedure for heterogeneous PRS cohorts with quantitative phenotypes.** In lieu of explicit case/control labels by which to generate heterogeneous cohorts, we define 'cases' as individuals whose phenotypes are a function of their PRSs, whereas 'controls' have phenotypes that are sampled completely randomly from the same distribution.
