# Supplementary material for: Leveraging correlations between variants in polygenic risk scores to detect heterogeneity in GWAS cohorts
Source: PLoS Genet. 2020 Sep 21;16(9):e1009015. doi: 10.1371/journal.pgen.1009015 (PMC7529195; doi:10.1371/journal.pgen.1009015)
Supplement: S4 Text — This weight function is applied over individuals according to their phenotypes as a percentile of the phenotype distribution in the domain [0, 1], and determines the contribution of individuals to the weighted correlation substitution in CLiP-Y. Optimization is performed over a small set of discovery simulated cohorts, and validated in a larger set of simulated cohorts. A set of candidate functions which pass a fixed threshold are stored, and of these the function which performs best on the larger validation set is selected. (PDF) [file pgen.1009015.s004.pdf]

```
Function PolynomialWeightSearch
Input:
deg; // Polynomial degree
N; // Size of the case/control cohort to simulate
k_disc = 5, k_val = 20; // Number sample cohorts to simulate for discovery, validation steps
S_thresh; // Threshold score for saving candidate coefficient
coef_sd; // std. dev. for random increments in coefficient search
Output: coefs
Function CalScore({X_het}, {X_hom}, coefs):
    return Mean(CLiP-Y({X_het}, coefs)) − Mean(CLiP-Y({X_hom}, coefs));
Function DiscoverCandidatePolynomials({X_het}, {X_hom}, S_thresh):
    cands = {} for Num candidates desired do
        coefs = ℝ_(deg+1) ∼ N(0, coef_sd);
        S_het = CalcScore({X_het}, {X_hom}, coefs);
        while Not converged do
            increment a randomly selected coefficient in coefs by a ∼ N(0, coef_sd);
            if CalcScore({X_het}, {X_hom}, coefs) > S_het then
                update coefs and S_het
            end
        end
        if S_het > S_thresh then
            store coefs in cands
        end
    end
    return cands
/* Simulate cohorts for discovery/validation */
{X_het}_disc = {X_1, ..., X_{k_disc} | X_i = SampleCLiP-Y(N, cohort = het)};
{X_het}_disc = {X_1, ..., X_{k_disc} | X_i = SampleCLiP-Y(N, cohort = hom)};
cands = DiscoverCandidatePolynomials({X_het}_disc, {X_hom}_disc, S_thresh);
{X_het}_val = {X_1, ..., X_{k_val} | X_i = SampleCLiP-Y(N, cohort = het)};
{X_het}_val = {X_1, ..., X_{k_val} | X_i = SampleCLiP-Y(N, cohort = hom)};
return argmax_{coefs∈cands}(CalcScore({X_het}_val, {X_hom}_val, coefs))
```

S4 Text. **Local search for optimal polynomial weight function ϕ for use with CLiP-Y.** This weight function is applied over individuals according to their phenotypes as a percentile of the phenotype distribution in the domain $[0, 1]$, and determines the contribution of individuals to the weighted correlation substitution in CLiP-Y. Optimization is performed over a small set of discovery simulated cohorts, and validated in a larger set of simulated cohorts. A set of candidate functions which pass a fixed threshold are stored, and of these the function which performs best on the larger validation set is selected.
