# Supplementary material for: Leveraging correlations between variants in polygenic risk scores to detect heterogeneity in GWAS cohorts
Source: PLoS Genet. 2020 Sep 21;16(9):e1009015. doi: 10.1371/journal.pgen.1009015 (PMC7529195; doi:10.1371/journal.pgen.1009015)
Supplement: S1 Fig — Each sample comprises 100,000 individuals with randomly sampled genotypes, and case/control status was assigned according to either a logistic or liability threshold model with a desired prevalence of 0.01. The resulting fraction of individuals assigned to cases by each model is plotted. Colors indicate a fixed odds ratio assigned to 10 SNPs, with each value simulated over 10 trials. (PDF) [file pgen.1009015.s005.pdf]

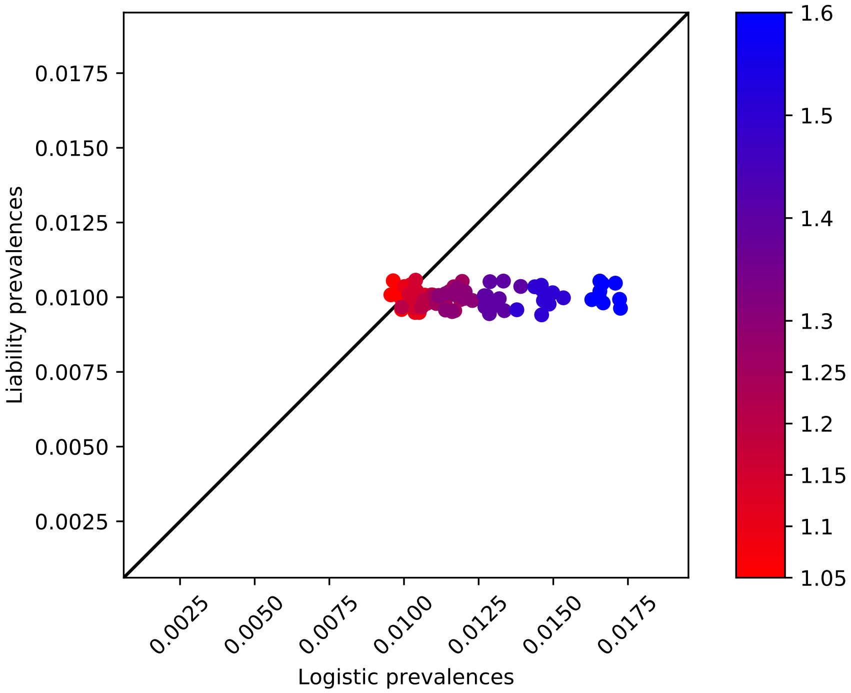

S1 Fig. **Sample disease prevalences of cohorts simulated according to logistic and liability threshold models.** Each sample comprises 100,000 individuals with randomly sampled genotypes, and case/control status was assigned according to either a logistic or liability threshold model with a desired prevalence of 0.01. The resulting fraction of individuals assigned to cases by each model is plotted. Colors indicate a fixed odds ratio assigned to 10 SNPs, with each value simulated over 10 trials.
