# Supplementary material for: Leveraging correlations between variants in polygenic risk scores to detect heterogeneity in GWAS cohorts
Source: PLoS Genet. 2020 Sep 21;16(9):e1009015. doi: 10.1371/journal.pgen.1009015 (PMC7529195; doi:10.1371/journal.pgen.1009015)
Supplement: S2 Fig — (A) Case/control cohorts generated from logistic or Risch models with 100 diploid SNPs with allele frequency of 0.2 and odds ratio of 1.2. A prior prevalence of 0.01 is assumed for both models. Scores of logistic cohorts exhibit a negative bias resulting from correlations between SNPs not seen in the Risch model. (B) Case/control cohorts generated from logistic or Risch models with 100 diploid SNPs with allele frequency of 0.2 and odds ratios of 1.06, and prior prevalence of 0.01. With smaller effect sizes, the negative bias in the logistic model disappears. (PDF) [file pgen.1009015.s006.pdf]

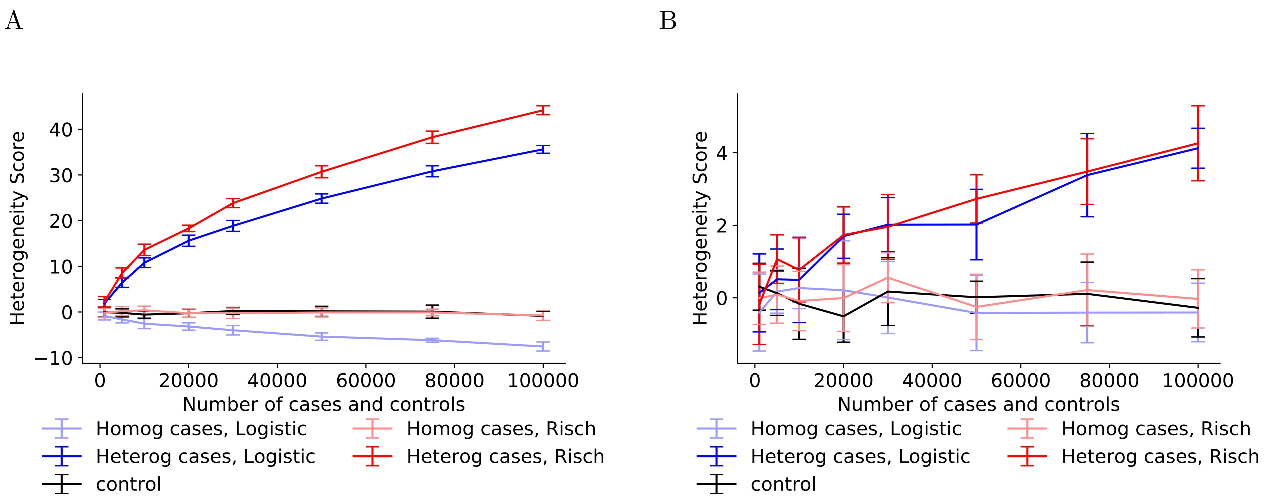

S2 Fig. **CLiP performance on simulated control, homogeneous, and heterogeneous cohorts.** Shown are heterogeneity scores resulting from different cohort sizes and genetic architectures. **(A)** Case/control cohorts generated from logistic or Risch models with 100 diploid SNPs with allele frequency of 0.2 and odds ratio of 1.2. A prior prevalence of 0.01 is assumed for both models. Scores of logistic cohorts exhibit a negative bias resulting from correlations between SNPs not seen in the Risch model. **(B)** Case/control cohorts generated from logistic or Risch models with 100 diploid SNPs with allele frequency of 0.2 and odds ratios of 1.06, and prior prevalence of 0.01. With smaller effect sizes, the negative bias in the logistic model disappears.
