# Supplementary material for: Leveraging correlations between variants in polygenic risk scores to detect heterogeneity in GWAS cohorts
Source: PLoS Genet. 2020 Sep 21;16(9):e1009015. doi: 10.1371/journal.pgen.1009015 (PMC7529195; doi:10.1371/journal.pgen.1009015)
Supplement: S3 Fig — Heterogeneity scores (y-axis) evaluated on heterogeneous cohorts comprising a mixture of true cases and controls at different proportions (x-axis). Colors indicate the total cohort size. The X-axis indicates the fraction of individuals that are true cases. When the fraction is 0, the cohort contains only controls, and all expected correlations are 0, producing a heterogeneity score of 0. When the fraction is 1, the cohort contains only cases, and produces a highly negative score due to negative correlations between all pairs of SNPs. As expected, a mixture of cases and controls produces positive scores, with the peak score occurring when the cohort is split evenly. More detailed results of this set of simulations are shown in S2 Table. All tests were conducted with a SNP variance explained of 0.05. (PDF) [file pgen.1009015.s007.pdf]

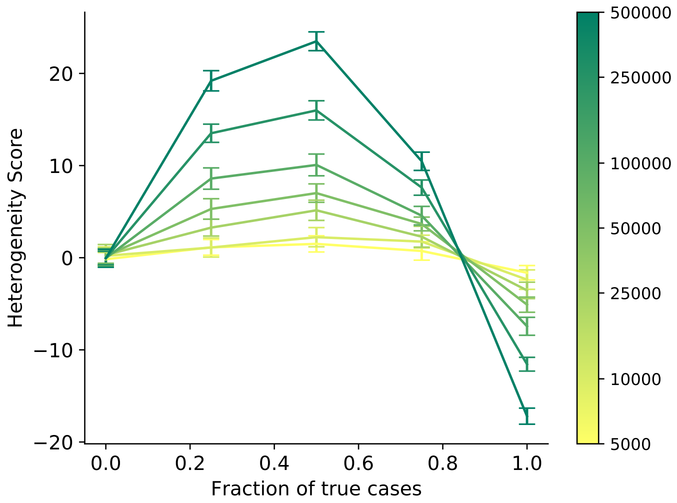

S3 Fig. **CLiP performance as a function of subtype fraction size.** Heterogeneity scores (y-axis) evaluated on heterogeneous cohorts comprising a mixture of true cases and controls at different proportions (x-axis). Colors indicate the total cohort size. The X-axis indicates the fraction of individuals that are true cases. When the fraction is 0, the cohort contains only controls, and all expected correlations are 0, producing a heterogeneity score of 0. When the fraction is 1, the cohort contains only cases, and produces a highly negative score due to negative correlations between all pairs of SNPs. As expected, a mixture of cases and controls produces positive scores, with the peak score occurring when the cohort is split evenly. More detailed results of this set of simulations are shown in 2 Table. All tests were conducted with a SNP variance explained of 0.05.
