# Supplementary material for: Leveraging correlations between variants in polygenic risk scores to detect heterogeneity in GWAS cohorts
Source: PLoS Genet. 2020 Sep 21;16(9):e1009015. doi: 10.1371/journal.pgen.1009015 (PMC7529195; doi:10.1371/journal.pgen.1009015)
Supplement: S4 Fig — CLiP corrects for confounding heterogeneity such as ancestry by calculating the difference in SNP-SNP correlations between cases and controls. Any confounding patterns that are present in controls as well as cases are then canceled from the score. (A) Simulated homogeneous and heterogeneous cohorts in which both cases and controls are sampled from two sub-populations with an Fst specified by color. (B) Power (dotted) and specificity (solid) over 20 trials. At high Fst values, both homogeneous and heterogeneous case scores are attenuated towards zero, and while power remains high, specificity begins to decline with values of Fst greater than 0.05. (PDF) [file pgen.1009015.s008.pdf]

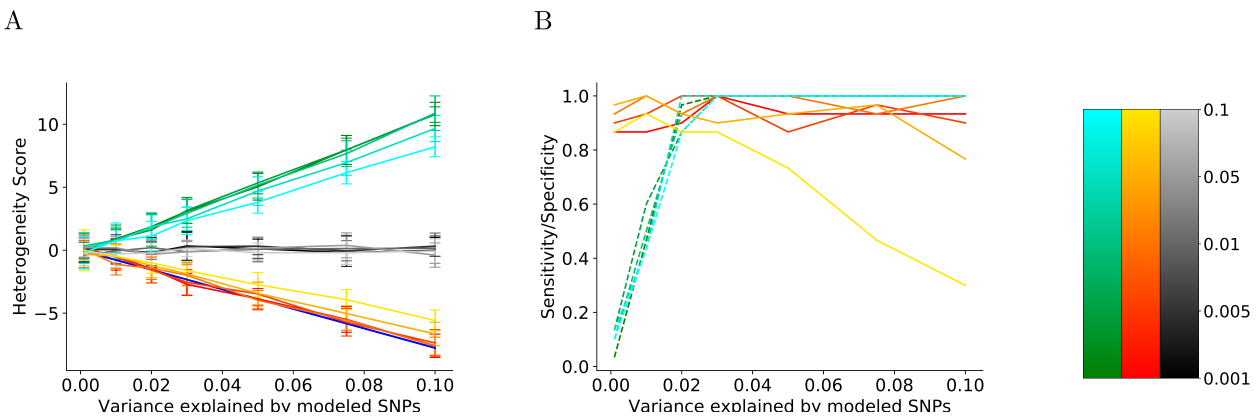

S4 Fig. **CLiP is robust to confounding heterogeneity with an $F_{st} < 0.05$.** CLiP corrects for confounding heterogeneity such as ancestry by calculating the difference in SNP-SNP correlations between cases and controls. Any confounding patterns that are present in controls as well as cases are then canceled from the score. **(A)** Simulated homogeneous and heterogeneous cohorts in which both cases and controls are sampled from two sub-populations with an F_{st} specified by color. **(B)** Power (dotted) and specificity (solid) over 20 trials. At high F_{st} values, both homogeneous and heterogeneous case scores are attenuated towards zero, and while power remains high, specificity begins to decline with values of F_{st} greater than 0.05.
