# Supplementary material for: Leveraging correlations between variants in polygenic risk scores to detect heterogeneity in GWAS cohorts
Source: PLoS Genet. 2020 Sep 21;16(9):e1009015. doi: 10.1371/journal.pgen.1009015 (PMC7529195; doi:10.1371/journal.pgen.1009015)
Supplement: S5 Fig — Performance is shown a function of the fraction of SNP effects unique to a particular sub-phenotype (x-axis). Colors indicate the number of sub-phenotypes. Simulations were performed with 50,000 simulated cases and 50,000 controls, and a total SNP variance explained over all sub-phenotype PRSs set to 0.05. More detailed results of this set of simulations are shown in S3 Table. (PDF) [file pgen.1009015.s009.pdf]

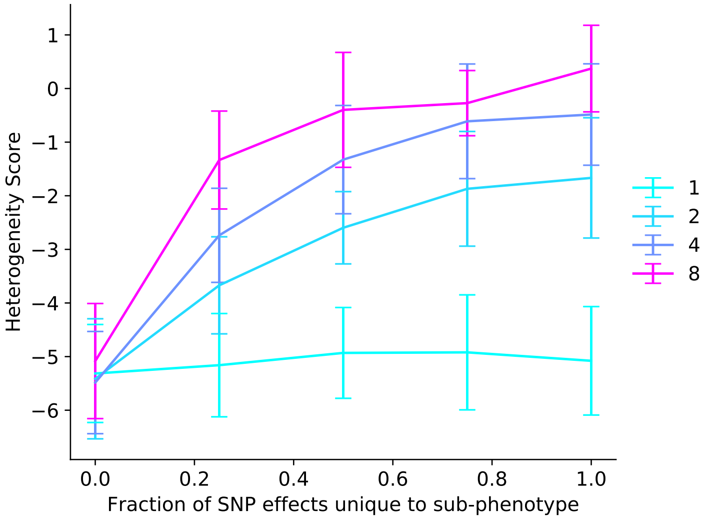


S5 Fig. **CLiP performance as a function of number of distinct subtypes.** Heterogeneity scores (y-axis) evaluated on simulated heterogeneous cohorts with disjoint sub-phenotypes. Performance is shown a function of the fraction of SNP effects unique to a particular sub-phenotype (x-axis). Colors indicate the number of sub-phenotypes. Simulations were performed with 50,000 simulated cases and 50,000 controls, and a total SNP variance explained over all sub-phenotype PRSs set to 0.05. More detailed results of this set of simulations are shown in 3 Table.
