# Supplementary material for: Leveraging correlations between variants in polygenic risk scores to detect heterogeneity in GWAS cohorts
Source: PLoS Genet. 2020 Sep 21;16(9):e1009015. doi: 10.1371/journal.pgen.1009015 (PMC7529195; doi:10.1371/journal.pgen.1009015)
Supplement: S6 Fig — To demonstrate that optimal quantitative weight functions for heterogeneity are concave functions, two interval indicator functions in [0, 1], an increasing one for [x, 1] (x-axis) and a decreasing one for [0, y] (y-axis) are combined so that their sum is the tested weight function. Each bin on the axes represents a transition point for the two step functions. The heterogeneity score is tested against a single homogeneous cohort, so optimal scores should be those that are most negative. (A) The best scores are those where x is low on the PRS percentile scale but not 0, while y is high on the PRS percentile scale but not 1. This coincides with the optimal polynomial functions obtained by a local search. (B) A zoomed view of the top left in A, showing that the optimal scores are not obtained by step functions at the periphery of the PRS distribution. (PDF) [file pgen.1009015.s010.pdf]

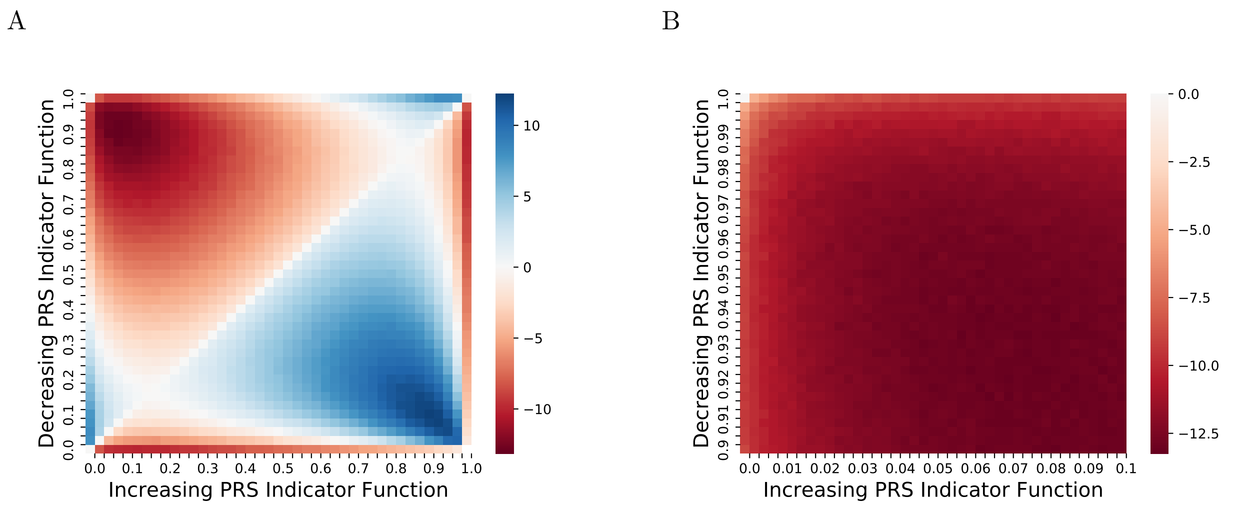

S6 Fig. **Creating CliP-Y weight functions using step functions.** To demonstrate that optimal quantitative weight functions for heterogeneity are concave functions, two interval indicator functions in $[0, 1]$, an increasing one for $[x, 1]$ (x-axis) and a decreasing one for $[0, y]$ (y-axis) are combined so that their sum is the tested weight function. Each bin on the axes represents a transition point for the two step functions. The heterogeneity score is tested against a single homogeneous cohort, so optimal scores should be those that are most negative. **(A)** The best scores are those where x is low on the PRS percentile scale but not 0, while y is high on the PRS percentile scale but not 1. This coincides with the optimal polynomial functions obtained by a local search. **(B)** A zoomed view of the top left in A, showing that the optimal scores are not obtained by step functions at the periphery of the PRS distribution.
