# Supplementary material for: Leveraging correlations between variants in polygenic risk scores to detect heterogeneity in GWAS cohorts
Source: PLoS Genet. 2020 Sep 21;16(9):e1009015. doi: 10.1371/journal.pgen.1009015 (PMC7529195; doi:10.1371/journal.pgen.1009015)
Supplement: S7 Fig — Means and standard deviations of scores are shown as a function of sample size: (A) homogeneous cohorts, (B) heterogeneous cohorts, and (C) the difference (heterogeneous minus homogeneous) scores. The color gradient indicates the location of the threshold separating cases and controls. Each condition was run for 20 trials, and all cohorts were simulated with 100 SNPs and a total variance explained of 0.1. (PDF) [file pgen.1009015.s011.pdf]

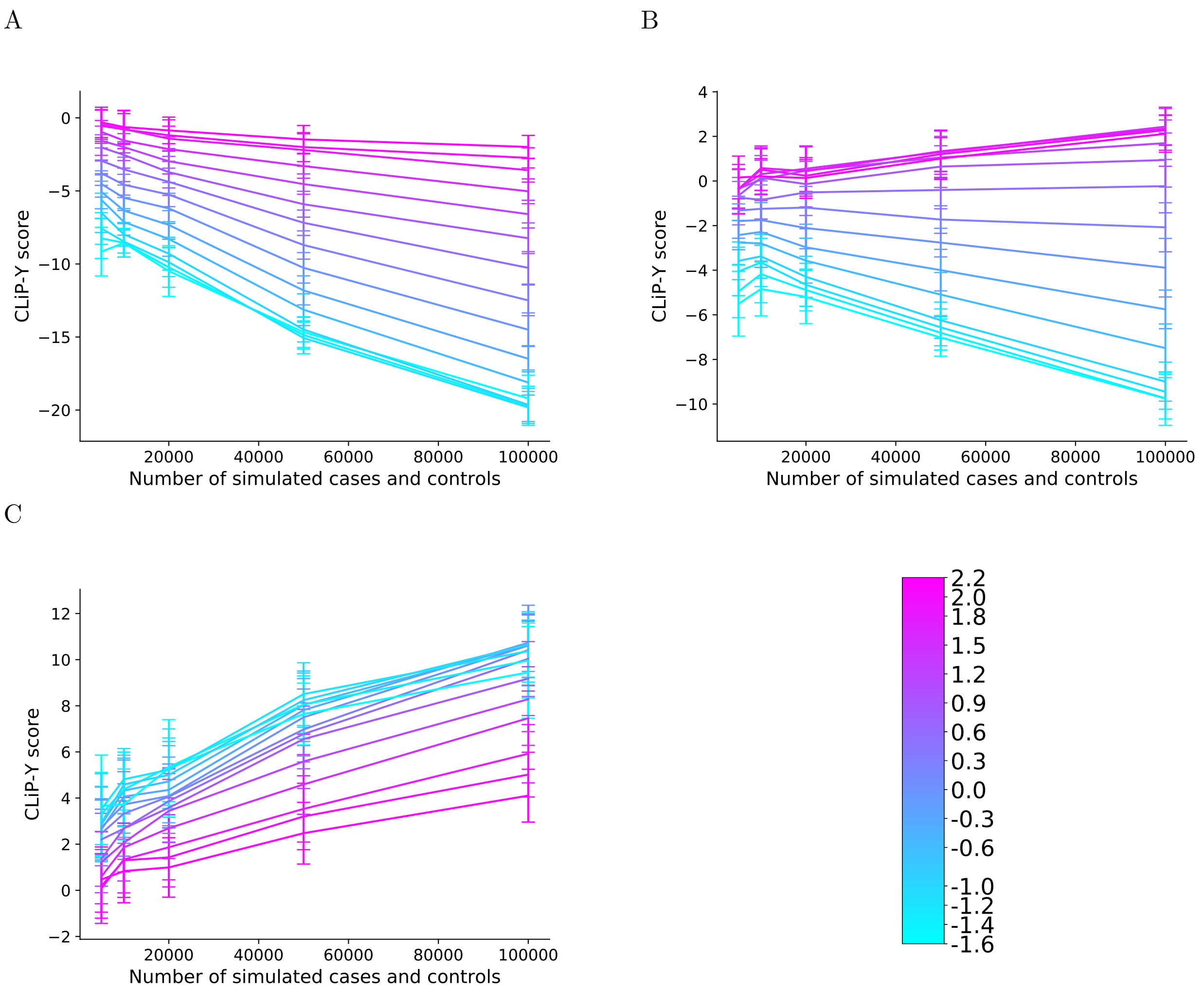

S7 Fig. **CLiP-Y scores for quantitative phenotypes split into artificial cases and controls by a hard threshold, as a function of cohort size.** Means and standard deviations of scores are shown as a function of sample size: **(A)** homogeneous cohorts, **(B)** heterogeneous cohorts, and **(C)** the difference (heterogeneous minus homogeneous) scores. The color gradient indicates the location of the threshold separating cases and controls. Each condition was run for 20 trials, and all cohorts were simulated with 100 SNPs and a total variance explained of 0.1.
