# Supplementary material for: Leveraging correlations between variants in polygenic risk scores to detect heterogeneity in GWAS cohorts
Source: PLoS Genet. 2020 Sep 21;16(9):e1009015. doi: 10.1371/journal.pgen.1009015 (PMC7529195; doi:10.1371/journal.pgen.1009015)
Supplement: S8 Fig — Means and standard deviations of scores are shown as a function of total variance explained by SNPs: (A) homogeneous cohorts, (B) heterogeneous cohorts, and (C) the difference between scores of heterogeneous cohorts and expected homogeneous scores in A. Colors indicate the type of weight function, with blue lines indicating learned polynomial functions. Each condition was run for 20 trials, and all cohorts were simulated with 100 SNPs and a sample size of 100,000 cases and 100,000 controls. (PDF) [file pgen.1009015.s012.pdf]

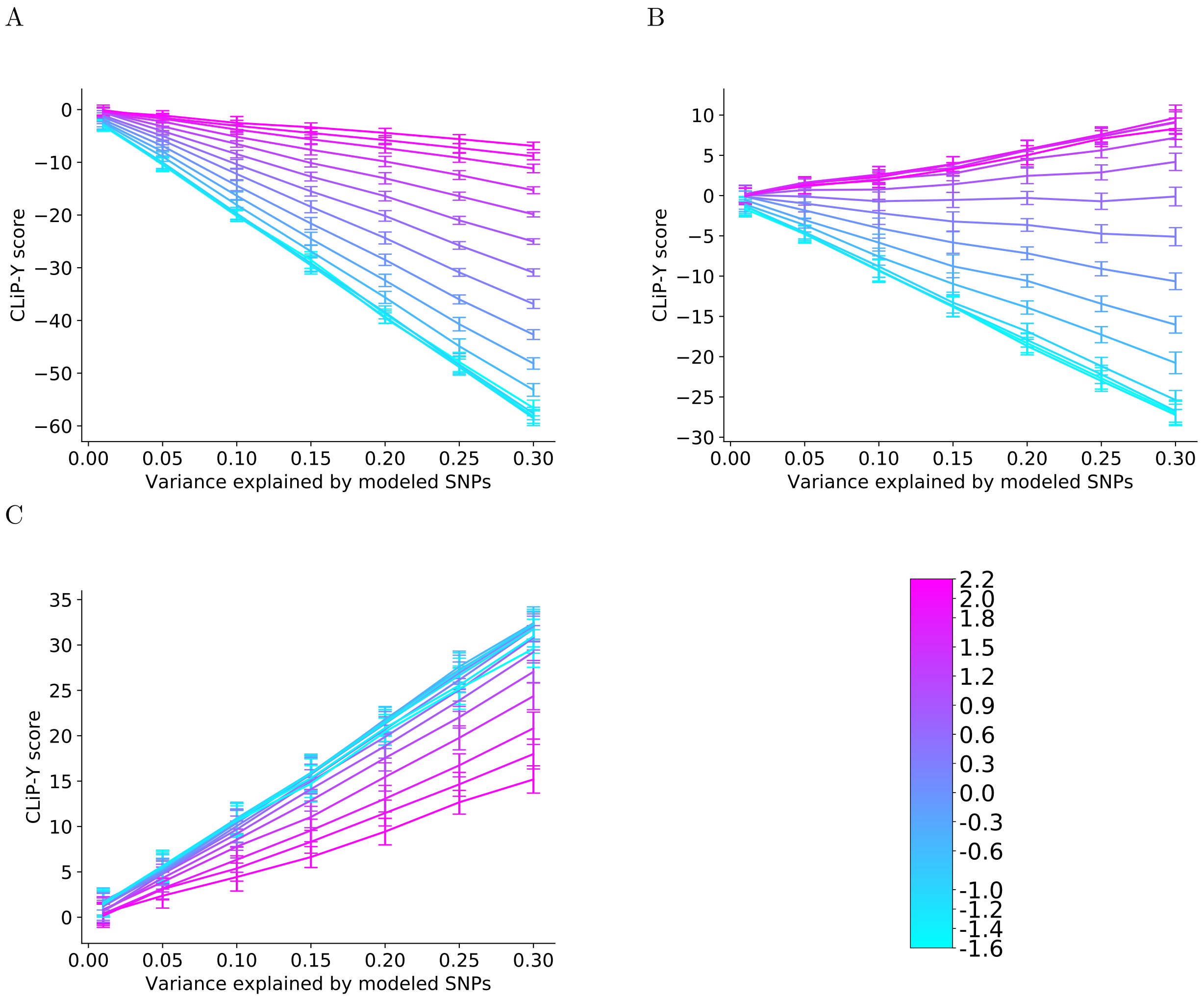

S8 Fig. **CLiP-Y scores for quantitative phenotypes split into artificial cases and controls by a hard threshold, as a function of SNP variance explained.** Means and standard deviations of scores are shown as a function of total variance explained by SNPs: **(A)** homogeneous cohorts, **(B)** heterogeneous cohorts, and **(C)** the difference between scores of heterogeneous cohorts and expected homogeneous scores in A. Colors indicate the type of weight function, with blue lines indicating learned polynomial functions. Each condition was run for 20 trials, and all cohorts were simulated with 100 SNPs and a sample size of 100,000 cases and 100,000 controls.
