# Supplementary material for: Leveraging correlations between variants in polygenic risk scores to detect heterogeneity in GWAS cohorts
Source: PLoS Genet. 2020 Sep 21;16(9):e1009015. doi: 10.1371/journal.pgen.1009015 (PMC7529195; doi:10.1371/journal.pgen.1009015)
Supplement: S9 Fig — Means and standard deviations of scores are shown as a function of sample size: (A) homogeneous cohorts, (B) heterogeneous cohorts, and (C) the difference between scores of heterogeneous cohorts and expected homogeneous scores in A. Colors indicate the type of weight function, with blue lines indicating learned polynomial functions. In panel A, black dotted lines indicate calculated expected scores for each homogeneous cohort, given effect sizes and allele frequencies. In practice, these expected scores will serve as the null scores subtracted from that of test data to produce analogous results to panel C. Each condition was run for 20 trials, and all cohorts were simulated with 100 SNPs and a total variance explained of 0.1. (PDF) [file pgen.1009015.s013.pdf]

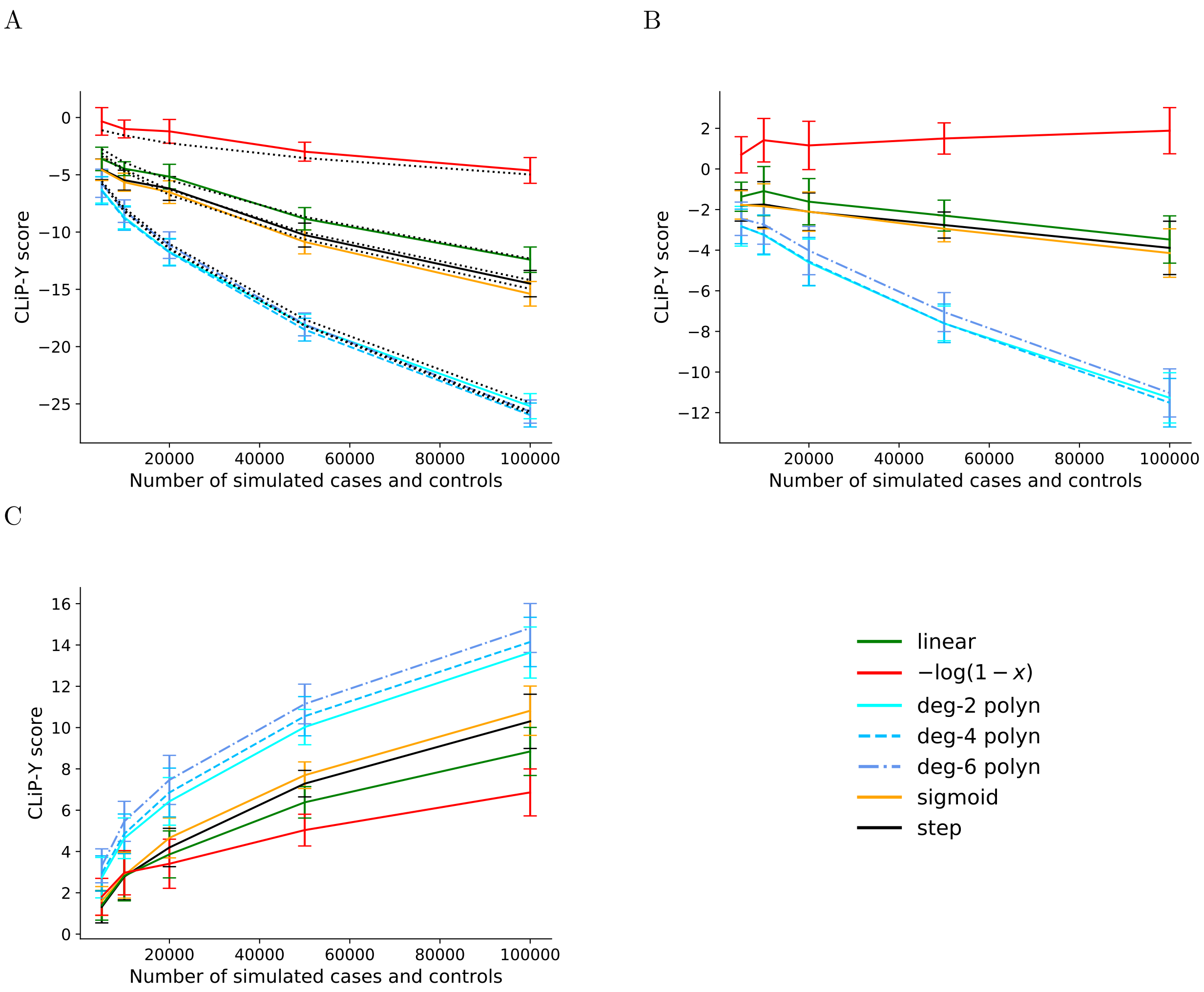

S9 Fig. **CLiP-Y scores for quantitative phenotypes with weight functions over individuals contributing to the correlation, as a function of cohort size.** Means and standard deviations of scores are shown as a function of sample size: **(A)** homogeneous cohorts, **(B)** heterogeneous cohorts, and **(C)** the difference between scores of heterogeneous cohorts and expected homogeneous scores in A. Colors indicate the type of weight function, with blue lines indicating learned polynomial functions. In panel A, black dotted lines indicate calculated expected scores for each homogeneous cohort, given effect sizes and allele frequencies. In practice, these expected scores will serve as the null scores subtracted from that of test data to produce analogous results to panel C. Each condition was run for 20 trials, and all cohorts were simulated with 100 SNPs and a total variance explained of 0.1.
