# Supplementary material for: Leveraging correlations between variants in polygenic risk scores to detect heterogeneity in GWAS cohorts
Source: PLoS Genet. 2020 Sep 21;16(9):e1009015. doi: 10.1371/journal.pgen.1009015 (PMC7529195; doi:10.1371/journal.pgen.1009015)
Supplement: S11 Fig — Shown are controls (black), homogeneous cases (red), and heterogeneous cases (green) across different levels of variance explained by generated predictors (VE2). The predicted score for homogeneous cases (blue), calculated from summary statistics used to generate predictors from sampled SNPs and phenotypes from predictors, is the true null hypothesis of the heterogeneity test. Means and standard errors are shown for 20 trials over 10 expression variables generated by 100 SNPs. (PDF) [file pgen.1009015.s015.pdf]

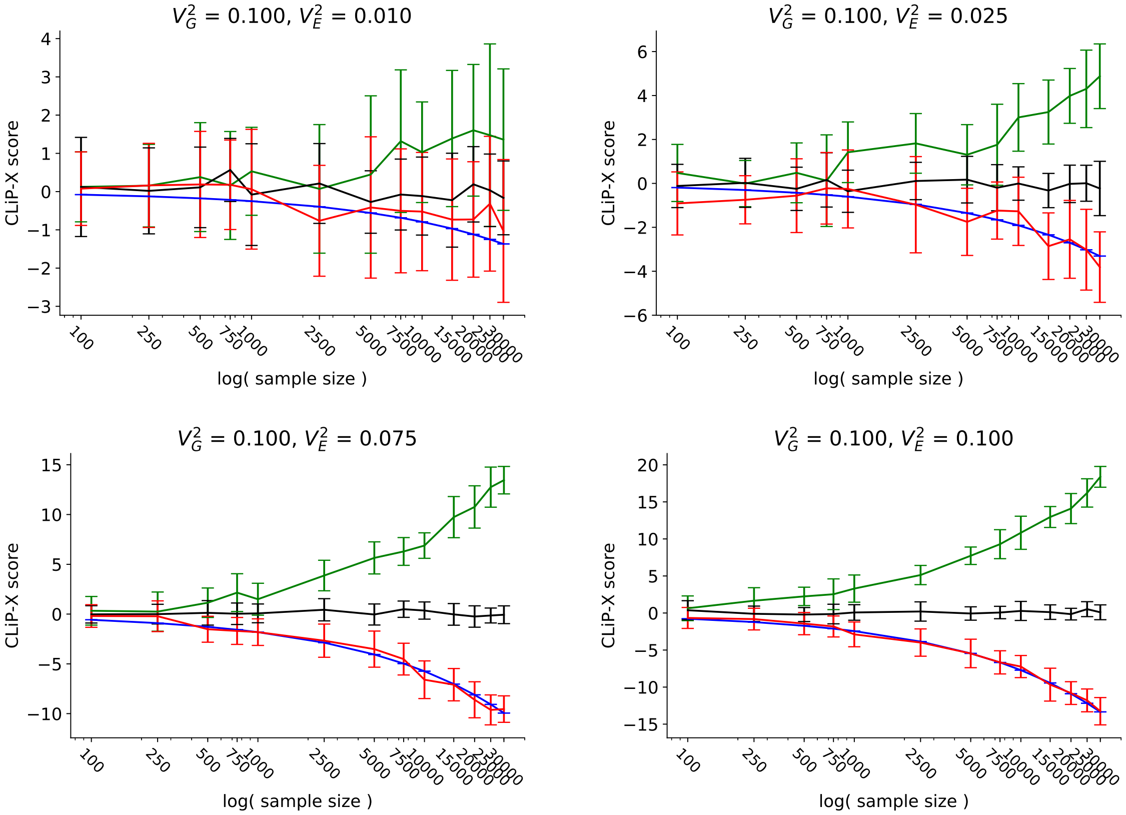

S11 Fig. **Additional CLiP-X scores as a function of variance explained by quantitative predictors.** Shown are controls (black), homogeneous cases (red), and heterogeneous cases (green) across different levels of variance explained by generated predictors (V_E^2). The predicted score for homogeneous cases (blue), calculated from summary statistics used to generate predictors from sampled SNPs and phenotypes from predictors, is the true null hypothesis of the heterogeneity test. Means and standard errors are shown for 20 trials over 10 expression variables generated by 100 SNPs.
