# Supplementary material for: Leveraging correlations between variants in polygenic risk scores to detect heterogeneity in GWAS cohorts
Source: PLoS Genet. 2020 Sep 21;16(9):e1009015. doi: 10.1371/journal.pgen.1009015 (PMC7529195; doi:10.1371/journal.pgen.1009015)
Supplement: S12 Fig — Shown are the average standard deviations between predicted and generated correlations as a function of the case sample size used to estimate the correlation. Predicted values account for contributions from PRS thresholding and shared SNP-expression effects. Standard deviations across all pairs of variables are averaged for each experiment. The black line denotes the function 1N-3, the expected standard deviation of the Fisher transformation for sample correlations. Values on the y-axis are transformed by an inverse hyperbolic function (artanh) for comparison to the Fisher transformation. (PDF) [file pgen.1009015.s016.pdf]

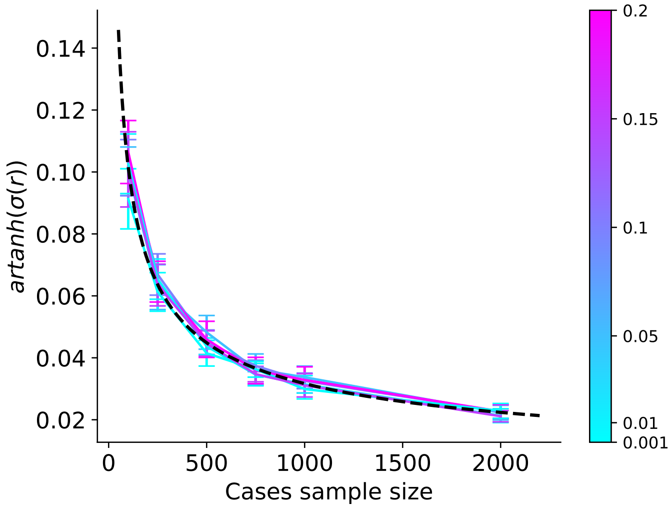

S12 Fig. **Validation of expected correlations in CLiP-X between expression variables in cases.** Shown are the average standard deviations between predicted and generated correlations as a function of the case sample size used to estimate the correlation. Predicted values account for contributions from PRS thresholding and shared SNP-expression effects. Standard deviations across all pairs of variables are averaged for each experiment. The black line denotes the function $\frac{1}{\sqrt{N-3}}$, the expected standard deviation of the Fisher transformation for sample correlations. Values on the y-axis are transformed by an inverse hyperbolic function (artanh) for comparison to the Fisher transformation.
