# Supplementary material for: Leveraging correlations between variants in polygenic risk scores to detect heterogeneity in GWAS cohorts
Source: PLoS Genet. 2020 Sep 21;16(9):e1009015. doi: 10.1371/journal.pgen.1009015 (PMC7529195; doi:10.1371/journal.pgen.1009015)
Supplement: S13 Fig — Original whole-cohort scores are shown in black. Ancestry inference was performed using fastStructure with number of ancestry groups k = 2 (red), k = 3 (green), and k = 4 (blue). While subgroups of small size are subject to larger correlation error, generally the heterogeneity scores of ancestry subgroups achieves the same magnitude as the original score, suggesting ancestry stratification is not the primary source of heterogeneity in PGC schizophrenia cohorts. (PDF) [file pgen.1009015.s017.pdf]

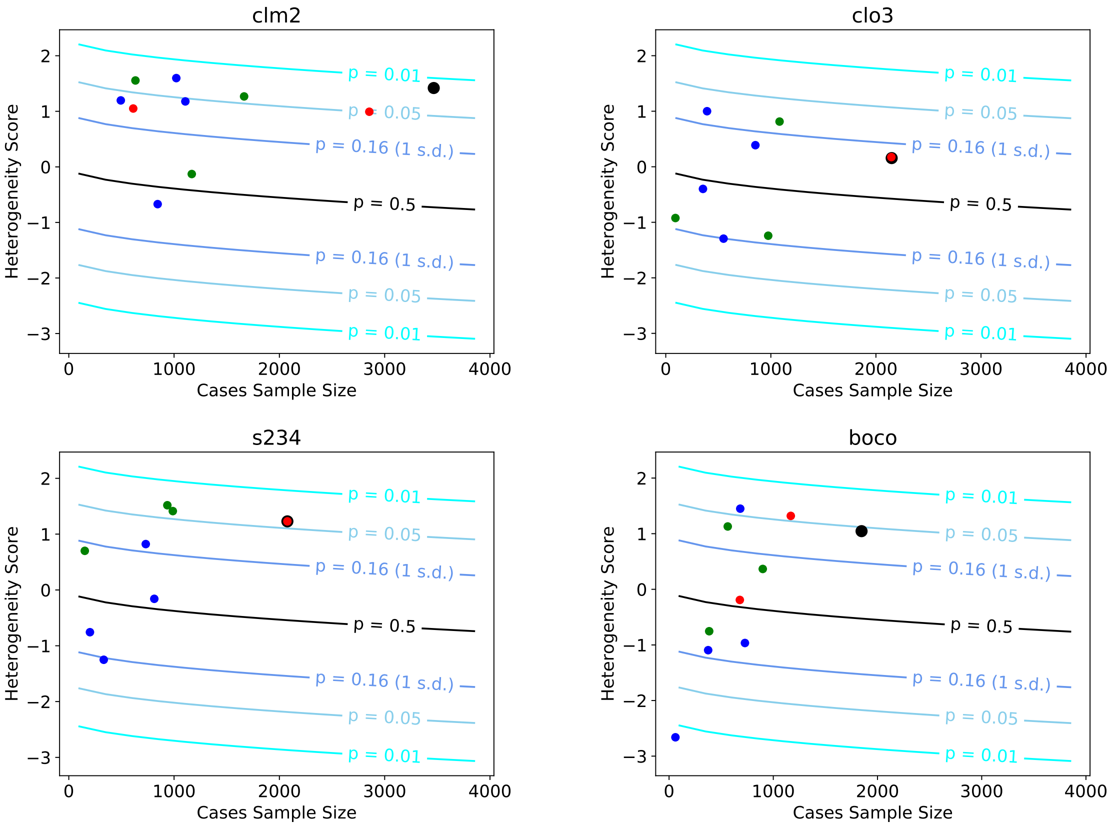

S13 Fig. **Heterogeneity scores of the largest PGC cohorts subdivided by inferred ancestry group.** Original whole-cohort scores are shown in black. Ancestry inference was performed using fastStructure with number of ancestry groups $k = 2$ (red), $k = 3$ (green), and $k = 4$ (blue). While subgroups of small size are subject to larger correlation error, generally the heterogeneity scores of ancestry subgroups achieves the same magnitude as the original score, suggesting ancestry stratification is not the primary source of heterogeneity in PGC schizophrenia cohorts.
