# Supplementary material for: Leveraging correlations between variants in polygenic risk scores to detect heterogeneity in GWAS cohorts
Source: PLoS Genet. 2020 Sep 21;16(9):e1009015. doi: 10.1371/journal.pgen.1009015 (PMC7529195; doi:10.1371/journal.pgen.1009015)
Supplement: S14 Fig — Because in most scenarios the inputs will represent gene expression, we simulated transcripts as linear functions of randomly sampled genotypes, allowing for prior correlations from single genotypes associated with multiple transcripts. To generate a desired number of cases (Red), batches of random transcripts are generated repeatedly, and those individuals whose liability scores pass the threshold T are concatenated to a growing list of cases. Heterogeneous cases are created by concatenating true cases with controls. (PDF) [file pgen.1009015.s018.pdf]

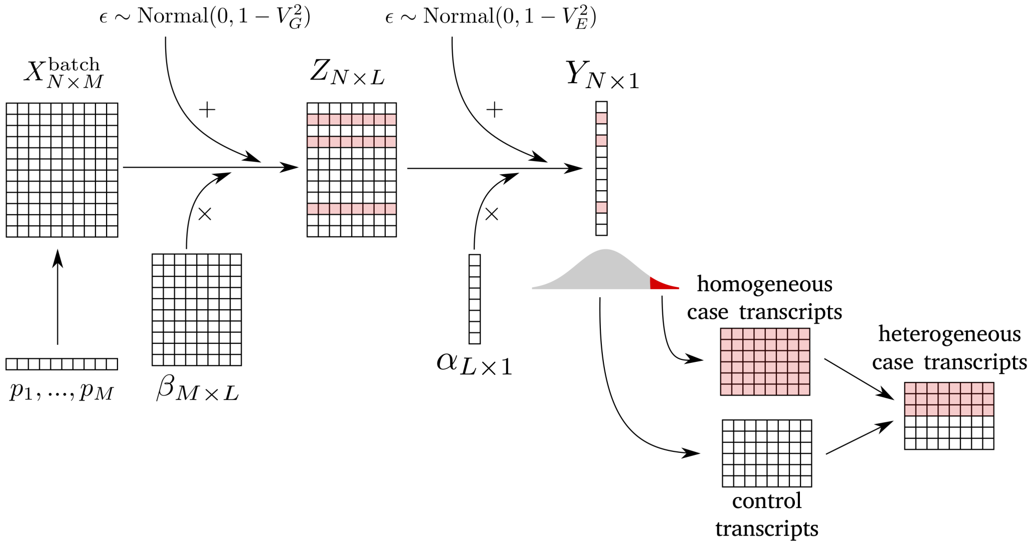

S14 Fig. **Sampling procedure for CLiP-X: heterogeneity detection with quantitative input variables.** Because in most scenarios the inputs will represent gene expression, we simulated transcripts as linear functions of randomly sampled genotypes, allowing for prior correlations from single genotypes associated with multiple transcripts. To generate a desired number of cases (Red), batches of random transcripts are generated repeatedly, and those individuals whose liability scores pass the threshold T are concatenated to a growing list of cases. Heterogeneous cases are created by concatenating true cases with controls.
