# Supplementary material for: Leveraging correlations between variants in polygenic risk scores to detect heterogeneity in GWAS cohorts
Source: PLoS Genet. 2020 Sep 21;16(9):e1009015. doi: 10.1371/journal.pgen.1009015 (PMC7529195; doi:10.1371/journal.pgen.1009015)
Supplement: S1 Table — For both methods, the reported power is scored on true heterogeneous cases by a Z-test with mean set to the expected score of homogeneous cases. In BUHMBOX, the expected score is 0 in keeping with a multiplicative assumption across SNPs. In CLiP, the expected score is some negative value corresponding to the negative correlations between SNPs expected of cases selected from a liability threshold model, providing a boost in power. Tables A and B correspond to panels A and B of Fig 3, respectively. “Sample Power” refers to the fraction TruePos.FalseNeg.+TruePos. of 20 trials shown in Fig 3 which pass a 95% confidence interval threshold. “Expected Power” refers to the percentile of the sample distribution of true heterogeneous cases passing the 95% confidence interval threshold. (PDF) [file pgen.1009015.s019.pdf]

A

Case Sample Size	Sample Power CLiP	Expected Power CLiP	Sample Power BUHMBOX	Expected Power BUHMBOX
1000	0.00	0.08	0.00	0.03
5000	0.55	0.63	0.25	0.17
10000	1.00	0.99	0.60	0.61
20000	1.00	1.00	0.80	0.81
30000	1.00	1.00	1.00	0.95
50000	1.00	1.00	1.00	1.00

B

Variance Explained	Sample Power CLiP	Expected Power CLiP	Sample Power BUHMBOX	Expected Power BUHMBOX
0.001	0.10	0.08	0.10	0.07
0.01	0.50	0.42	0.05	0.13
0.02	1.00	0.99	0.60	0.61
0.03	1.00	1.00	0.90	0.96
0.05	1.00	1.00	1.00	1.00
0.075	1.00	1.00	1.00	1.00
0.1	1.00	1.00	1.00	1.00

S1 Table. **Power calculations accompanying CLiP and BUHMBOX simulation results in Fig 3.** For both methods, the reported power is scored on true heterogeneous cases by a Z-test with mean set to the expected score of homogeneous cases. In BUHMBOX, the expected score is 0 in keeping with a multiplicative assumption across SNPs. In CLiP, the expected score is some negative value corresponding to the negative correlations between SNPs expected of cases selected from a liability threshold model, providing a boost in power. Tables A and B correspond to panels A and B of Fig 3, respectively. "Sample Power" refers to the fraction $\frac{\text{True Pos.}}{\text{False Neg.}+\text{True Pos.}}$ of 20 trials shown in Fig 3 which pass a 95% confidence interval threshold. "Expected Power" refers to the percentile of the sample distribution of true heterogeneous cases passing the 95% confidence interval threshold.
