# Supplementary material for: Leveraging correlations between variants in polygenic risk scores to detect heterogeneity in GWAS cohorts
Source: PLoS Genet. 2020 Sep 21;16(9):e1009015. doi: 10.1371/journal.pgen.1009015 (PMC7529195; doi:10.1371/journal.pgen.1009015)
Supplement: S2 Table — These simulations differ by total variance explained (h2), cohort size, and percentage of individuals in the cohort that are true cases, with the remaining individuals being simulated controls. All trials were run with 100 SNPs with a fixed uniform effect size and an allele frequency of 0.2. Shown are mean and standard deviations of 20 trials. (PDF) [file pgen.1009015.s020.pdf]

# cases | % true		h^2 0.01	0.025	0.05	0.075	0.1
5000	0	0.21 ± 1.08	0.07 ± 0.85	-0.16 ± 0.72	-0.21 ± 0.91	-0.07 ± 0.79
	0.25	0.14 ± 0.64	0.18 ± 0.87	1.13 ± 0.85	1.30 ± 0.97	2.00 ± 0.80
	0.5	-0.02 ± 0.94	0.80 ± 0.98	1.50 ± 0.88	2.58 ± 1.11	3.87 ± 0.93
	0.75	0.11 ± 1.01	0.53 ± 0.90	0.73 ± 1.00	1.24 ± 1.17	1.49 ± 0.90
	1.0	-0.25 ± 0.88	-0.94 ± 0.94	-1.63 ± 0.78	-2.42 ± 0.91	-3.49 ± 1.05
10000	0	-0.08 ± 0.90	-0.20 ± 1.01	0.21 ± 0.97	0.41 ± 0.99	-0.11 ± 1.16
	0.25	-0.34 ± 0.62	0.63 ± 0.74	1.10 ± 1.03	3.01 ± 0.88	4.07 ± 0.84
	0.5	0.28 ± 0.83	1.07 ± 1.32	2.23 ± 1.04	4.17 ± 0.63	6.13 ± 0.99
	0.75	-0.02 ± 1.19	0.95 ± 1.23	1.75 ± 0.69	2.04 ± 0.88	2.63 ± 0.99
	1.0	-0.31 ± 0.74	-0.75 ± 1.11	-2.38 ± 1.05	-3.52 ± 1.04	-4.79 ± 1.04
25000	0	0.19 ± 1.08	-0.25 ± 0.99	0.30 ± 1.14	0.28 ± 0.94	0.12 ± 1.05
	0.25	0.82 ± 0.82	1.47 ± 0.74	3.27 ± 0.91	5.55 ± 1.15	7.33 ± 1.33
	0.5	0.43 ± 1.11	2.06 ± 0.98	5.15 ± 1.10	7.27 ± 0.92	9.73 ± 1.08
	0.75	0.59 ± 1.00	1.05 ± 0.97	2.28 ± 1.13	3.32 ± 0.94	4.72 ± 1.05
	1.0	-0.75 ± 0.84	-1.94 ± 1.19	-3.55 ± 0.89	-5.45 ± 1.00	-7.82 ± 0.83
50000	0	-0.23 ± 1.07	-0.12 ± 0.71	0.21 ± 0.81	0.43 ± 1.15	-0.15 ± 1.04
	0.25	0.56 ± 1.08	2.22 ± 0.78	5.29 ± 1.11	8.89 ± 1.36	11.78 ± 1.07
	0.5	1.15 ± 1.17	3.35 ± 0.93	7.01 ± 1.00	10.69 ± 1.28	13.86 ± 0.94
	0.75	0.95 ± 0.99	1.89 ± 0.83	3.66 ± 0.75	5.19 ± 0.76	6.40 ± 1.02
	1.0	-0.93 ± 1.06	-2.51 ± 1.10	-5.10 ± 0.82	-8.43 ± 0.99	-10.74 ± 0.96
100000	0	0.39 ± 0.94	-0.43 ± 1.02	-0.05 ± 0.80	0.15 ± 0.95	0.15 ± 0.95
	0.25	1.17 ± 0.99	3.50 ± 1.20	8.59 ± 1.16	12.78 ± 1.23	17.05 ± 0.84
	0.5	1.61 ± 1.09	5.20 ± 0.95	10.07 ± 1.18	15.31 ± 1.11	20.76 ± 1.07
	0.75	0.66 ± 0.74	2.42 ± 0.81	4.56 ± 1.01	7.10 ± 1.11	9.04 ± 1.08
	1.0	-1.48 ± 1.09	-3.68 ± 1.14	-7.44 ± 0.97	-11.21 ± 1.00	-15.05 ± 0.78
250000	0	-0.18 ± 0.99	0.15 ± 0.99	-0.04 ± 0.71	0.37 ± 0.89	-0.40 ± 1.04
	0.25	2.66 ± 0.83	6.36 ± 1.19	13.51 ± 0.99	21.10 ± 0.78	27.63 ± 0.85
	0.5	3.35 ± 1.13	8.29 ± 0.83	15.99 ± 1.04	24.57 ± 1.21	32.30 ± 1.03
	0.75	1.43 ± 0.82	3.79 ± 0.73	7.61 ± 0.83	10.94 ± 1.09	15.00 ± 1.15
	1.0	-2.10 ± 1.11	-5.79 ± 1.21	-11.56 ± 0.75	-18.16 ± 0.95	-24.52 ± 0.84
500000	0	0.51 ± 0.92	0.23 ± 0.70	-0.06 ± 0.97	-0.21 ± 0.75	0.04 ± 0.68
	0.25	3.47 ± 0.75	9.83 ± 0.93	19.20 ± 1.10	29.40 ± 0.91	39.72 ± 0.91
	0.5	4.40 ± 0.83	11.23 ± 1.45	23.49 ± 1.02	34.76 ± 0.86	45.98 ± 1.09
	0.75	2.36 ± 0.84	5.46 ± 0.99	10.47 ± 0.99	15.97 ± 0.85	21.10 ± 1.10
	1.0	-3.50 ± 0.88	-8.25 ± 0.85	-17.19 ± 0.87	-25.45 ± 1.00	-34.29 ± 1.05

S2 Table. **Simulated CLiP results over additional parameters.** These simulations differ by total variance explained (h^2), cohort size, and percentage of individuals in the cohort that are true cases, with the remaining individuals being simulated controls. All trials were run with 100 SNPs with a fixed uniform effect size and an allele frequency of 0.2. Shown are mean and standard deviations of 20 trials.
