# Supplementary material for: Leveraging correlations between variants in polygenic risk scores to detect heterogeneity in GWAS cohorts
Source: PLoS Genet. 2020 Sep 21;16(9):e1009015. doi: 10.1371/journal.pgen.1009015 (PMC7529195; doi:10.1371/journal.pgen.1009015)
Supplement: S3 Table — Entries comprise mean and standard deviations of CLiP scores evaluated over 20 trials, with a total variance explained of 0.05 and total case set size of 50000. All simulations were performed with 100 SNPs, and in independent sub-phenotypes these 100 were subdivided equally among the number of sub-phenotypes. The percentage of SNPs shared refers to the percentage of SNPs within each sub-phenotype which has a fixed effect size across all sub-phenotypes. (PDF) [file pgen.1009015.s021.pdf]

% SNPs shared / # sub-phenos	0	0.25	0.5	0.75	1
1	-5.08 ± 1.01	-4.92 ± 1.07	-4.93 ± 0.85	-5.16 ± 0.96	-5.32 ± 0.91
2	-1.67 ± 1.12	-1.87 ± 1.07	-2.60 ± 0.67	-3.67 ± 0.91	-5.41 ± 1.12
3	-0.65 ± 0.79	-1.23 ± 0.91	-1.98 ± 0.90	-2.90 ± 0.74	-4.99 ± 0.78
4	-0.49 ± 0.95	-0.61 ± 1.07	-1.33 ± 1.01	-2.74 ± 0.88	-5.48 ± 0.95
5	-0.56 ± 1.00	-0.81 ± 1.01	-0.97 ± 1.02	-2.00 ± 1.23	-5.53 ± 0.80
6	-0.44 ± 1.09	-0.39 ± 1.01	-1.14 ± 0.93	-2.05 ± 0.81	-5.07 ± 0.75
7	-0.05 ± 0.90	-0.12 ± 0.98	-0.43 ± 0.92	-1.68 ± 0.93	-5.42 ± 1.04
8	0.37 ± 0.81	-0.27 ± 0.61	-0.40 ± 1.07	-1.33 ± 0.91	-5.08 ± 1.07

S3 Table. **Simulated CLiP results using cases generated from multiple correlated sub-phenotypes.** Entries comprise mean and standard deviations of CLiP scores evaluated over 20 trials, with a total variance explained of 0.05 and total case set size of 50000. All simulations were performed with 100 SNPs, and in independent sub-phenotypes these 100 were subdivided equally among the number of sub-phenotypes. The percentage of SNPs shared refers to the percentage of SNPs within each sub-phenotype which has a fixed effect size across all sub-phenotypes.
