# Supplementary material for: Leveraging correlations between variants in polygenic risk scores to detect heterogeneity in GWAS cohorts
Source: PLoS Genet. 2020 Sep 21;16(9):e1009015. doi: 10.1371/journal.pgen.1009015 (PMC7529195; doi:10.1371/journal.pgen.1009015)
Supplement: Attachment — Submitted filename: PLOS_genetics_heterogeneity_comments.docx [file pgen.1009015.s022.docx]

Reviewer #1: The authors present a method to detect heterogeneity within a single GWAS sample. The main method (CLiP) is designed for case-control GWAS with SNP data, using only those SNPs that reach genome-wide significance in the GWAS to form a PRS. The authors explore two main scenarios: (1) misclassification in which the cases are comprised of true cases and controls, and (2) the cases are made up of subtypes of the diseases and controls are true controls for both subtypes. The simulations are well thought out and demonstrate the behavior of this method in a variety of scenarios. However, the authors do not spend adequate space discussing the benefit of CLiP versus prior methods with no direct comparisons of other metrics. Additionally, they do not demonstrate the distinction between multiple sources of heterogeneity, such as batch effects, population stratification, case misclassification, or clinical subtypes. This is a noted limitation of prior methods, therefore should be addressed in the main text.

We would like to greatly thank the reviewer for comments regarding comparison to previous methods, robustness of CLiP to batch effects, and distinguishing different types of heterogeneity. We hope to have addressed these points in our answers to specific comments below. Regarding comparison of CLiP to existing methods, the only direct comparison of performance we can make is to BUHMBOX, the method on which CLiP is based. Other methods described in the Introduction attempt to infer a full set of cluster-specific SNP effects, and these methods (matrix factorization and reverse GWAS) largely do not have sufficient power to perform this inference in single highly polygenic traits. CLiP exchanges this task for a simpler one of simply detecting heterogeneity, without fully distinguishing SNP effects, allowing for greater power for detection as well as correction for batch effects (described below). In our comparison to BUHMBOX, we have added p-values for scores taken from 0 as well as the negative values expected in CLiP (also described below).

Major comments:

• The Results section is currently written similarly to a Method section, with much of it detailing the simulation framework. While this is helpful, the section would also benefit from more context given to the results, with specific numbers given within the text. It is currently up to the reader to interpret the Results from the figures, which can be confusing.

Thank you for this suggestion. We have added additional text referencing the figures, especially for the case/control simulations in Figure 2 (page 11), as this is our first mention of the heterogeneity score plot as well as the simulation result directly applicable to the schizophrenia data set. The added text explains in more detail the difference in performance between the simulated cohorts and describes power calculations for heterogeneous cohorts, which are reported fully in the supplementary material.

• The major criticisms of prior methods in the Introduction cite the inability to distinguish the hidden factors as due to population stratification or batch effects. However, it is not clear that this method is able to distinguish from these reasons either, as the example from PGC shows that it can detect batch effects between cohorts (pooled analysis) that is not present in the summed stratified analyses.

Thank you for raising this issue. Like BUHMBOX, CLiP calculates the difference in summed correlations between cases and controls of a single cohort. If there exist background sources of heterogeneity such as population stratification, subtracting the control correlations would cancel out any confounding of the score if it is assumed that this heterogeneity is present in equal degree in both the sampled cases and controls. To confirm robustness of CLiP to this type of confounding heterogeneity, we have added analysis of simulated case/control cohorts with population stratification, described in the main text on page 10 and in supplementary figure S4. Briefly, we assume controls comprise equal proportions of two hidden sub-populations whose SNP allele frequencies differ by a desired Fst. Cases are sampled from controls as before, and are not guaranteed to comprise equal proportions of sub-populations. We find in figure S4 that while power remains robust over the range of Fst tested, specificity drops to 0.8 at Fst = 0.05. As we describe in the main text, this Fst is more descriptive of continent-level population differences and is far larger than what we would expect of a study conducted over a well-defined population in a single geographical area.

Batch effects between cohorts are a source of heterogeneity we have sought to correct by meta-analyzing per-cohort scores, but we believe the same issue does not apply to the same extent in the single cohorts themselves, as we describe in a paragraph added to the Methods section on meta-analyzing heterogeneity scores (page 38). Combining different cohorts not only combines individuals from populations that are more diverse than those collected within a single study, but does so in differing proportions due to differing sizes of the cohorts. The population proportions are then also expected to differ between the combined cases and controls, so the controls no longer represent the same background heterogeneity pattern as that of the cases.

• As CLiP is an extension of BUHMBOX, but correcting for the negative correlation bias, it would be helpful for a direct and explicit comparison, both in the simulations and the applied PGC example. For example, a figure showing the power to detect heterogeneity with and without this correction should be included in the main results.

This is an important point in light of the difference in expected correlation bias between the logistic and liability models: depending on which model is correct, the p-value should be taken from an expected value that is highly negative or near zero. In Table 1 displaying the heterogeneity scores of schizophrenia cohorts, we have now included the p-value taken from 0 in parentheses next to the original value. This would be the equivalent of applying BUHMBOX to scoring heterogeneity in single traits. Importantly, these values along with the score of all combined cohorts do not pass a p-value threshold of 0.05, indicating the choice of regression model plays a significant role in detecting heterogeneity. We have also added power calculations taken from 0 to the CLiP simulation results in both sub-figures of Figure 2. These results are shown in Table S1. Reported are both the sample power as a fraction of the number of simulated trials, and the power estimated from a normal distribution fitted over the set of sample heterogeneity scores.

• It is not clear from the manuscript if it is possible to distinguish between batch effects and heterogeneity by subtype/misclassification within a single sample. In the applied example given in PGC, multiple cohorts are required to distinguish between heterogeneity overall (pooled cases/controls) and heterogeneity without batch effects from the different cohorts (summed across cohort-level heterogeneity scores). Therefore, it may be misleading to conclude that this method is superior from other previous methods due to this ability.

We show in the added supplemental figure S4 that CLiP is robust to confounding heterogeneity that exists in both cases and controls of a single cohort as long as the confounding signal is not too large, such as an Fst larger than 0.05 (see our response to point 2 for more details). Similarly, we expect CLiP to be robust to modest amounts of confounding from other sources than ancestry, such as batch effects. In this respect we believe CLiP is more robust to background heterogeneity than previous mixture model approaches, which do not have an analogous capability to cancel signals in controls.

With respect to the magnitude of the confounding from batch effects, we expect the combination of multiple cohorts to contribute much more heterogeneity than that expected within individuals of a single cohort, simply because we are comparing individuals collected from many different geographical regions and likely different sub-populations rather than a single region. Batch effects from other sources such as sequencing error are also reduced as individuals within a single cohort are processed by the same lab.

Nevertheless, any detected signal cannot be directly attributed to etiological heterogeneity except by ruling out other sources such as demographics or ancestry, but doing so requires that these factors are observed. We have added text to the Discussion addressing caveats to detecting heterogeneity using CLiP, including this point. Any further efforts to rule out batch effects will likely involve more standardized data collection, or genotyping of more populations to test if the heterogeneity is consistently reproduced.

• The majority of simulations center around a misclassification framework, in which the “cases” are comprised of both true cases and controls. The framing of the method around subtypes, with examples given for bipolar disorder and schizophrenia, is not fully justified by the results and simulations. The manuscript may be better served by expanding upon results presented in Figure 2B to support this utility.

Thank you for raising this issue, as it has inspired some further clarification on how we expect our simulations to compare to heterogeneity in real disease cohorts. We chose to focus primarily on the misclassification scenario because it appears more generalizable than subtypes with mutually exclusive SNP effects and requires fewer assumptions, such as the number of subtypes and the partition of SNPs between them. While we label it misclassification, this scenario can also arise if a majority of known SNP associations pertain to one predominant subtype and not the rest, which can be interpreted as another form of etiologically-relevant subtyping. Additionally, misclassification is easier to detect because heterogeneity scores of the subtyping scenario are attenuated by negative correlations between SNPs belonging to different subtypes.

However, we wanted to address this issue further, including whether it is possible to differentiate between the two scenarios. First, while in the Methods we describe misclassification and subtyping as two forms of heterogeneity, we expect the true etiology of heterogeneous diseases to fall somewhere between these two extremes: rather than SNP associations being evenly partitioned between two subtypes such that the PRS quantifies risk equally in both subtypes, it is more likely that among known associated SNPs, associations to one subtype are stronger than those to another subtype. To simulate this, we assumed two subtypes with associated SNPs S1 and S2 and fixed effect size $\beta_{1}$and $\beta_{2}$, respectively. While we keep $\beta_{1}$ fixed, we vary $\beta_{2}$within the range from 0 to the magnitude of $\beta_{1}$. Thus when $\beta_{2}=0$, the SNPs in S2 are not ascertained and the SNPs S1 resemble the misclassification scenario; when $\beta_{2}=\beta_{1}$, the SNPs resemble the subtyping scenario. We have added a new figure (Figure 3) to illustrate this simulation, and we have moved the original Figure 2B to the supplementary material.

For any particular cohort, we cannot definitively distinguish between misclassification and subtyping, as the resultant score is dependent on many parameters including the number and magnitude of the SNP effects as well as the cohort size. But as we depict in Figure 3, we can distinguish qualitatively between these two scenarios based on the magnitude of the heterogeneity score prior to correction by the null. In Figure 3B, while both scenarios produce scores significantly larger than the negative expected score, the magnitude of the score is significantly positive under misclassification, and negative under subtyping. Under misclassification, any pair of SNPs is expected to produce a positive correlation, whereas under subtyping, correlations between SNPs belonging to different subtypes are negative, exactly canceling out the positive within-subtype correlations when $\beta_{2}=\beta_{1}$. The overall score is still negative, however, due to the negative correlation bias of the liability model overall SNPs. In contrast, Figure 3C depicts the same experiment conducted under the logistic model: as the negative correlation bias is largely absent, the subtyping scenario produces scores near 0 and are thus undetectable. Heterogeneity scores of the larger PGC cohorts are consistently positive, suggesting that the misclassification scenario is a closer culprit for heterogeneity than subtyping.

• The application of CLiP to a schizophrenia case-control GWAS could also be expanded. While subtypes of schizophrenia are less well defined, an exploration for possible sources of heterogeneity within these cohorts would be useful for interpretation. As subtypes of bipolar disorder are better defined, if this data is available it may be a better suited example to pick apart the potential contributions of batch effects, population stratification, and heterogeneity due to subtypes.

Thank you for this suggestion. We recently obtained access to the UK Biobank for the purpose of testing CLiP in more phenotypes. While we agree that bipolar disorder is a prime candidate for heterogeneity validation, unfortunately given the variance explained of known SNP associations and the sample size within the UK Biobank, we concluded we did not have sufficient power to detect heterogeneity within this trait. We did however perform a validation of CLiP-Y using neuroticism for the quantitative phenotype in a random sample of roughly 10,000 UK Biobank individuals. We detect heterogeneity with a p-value of 4.68e-9 (see Table 2), and though neuroticism has been reported to comprise two distinct subtypes scored from subsets of the neuroticism questionnaire, we found that partitioning the data on membership in these subtypes did not account for the observed heterogeneity in the entire cohort.

We also partitioned individuals in single PGC cohorts by inferred ancestry group assigned by fastStructure and scored these inferred clusters of individuals separately using CLiP. These results are shown in Supplementary Figure S7. We likewise did not find a significant reduction in heterogeneity in these subgroups in comparison to the entire cohorts. From these results it appears that heterogeneity is not easily decomposed across known covariates. Unfortunately we could not perform the same analysis over sex, as among the largest cohorts within the PGC data, sex information was either greatly imbalanced towards one sex or missing for certain cohorts.

• A discussion of the limitations for this method should be included in the Discussion. For example, what are the assumptions made for the negative correlation bias correction that may not be appropriate for some scenarios?

We have expanded the Discussion to include limitations of CLiP along with the difference between logistic and liability threshold expected scores. It is clear that the validity of the negative correlation bias is dependent on which model is true. We raise two arguments in support of the liability threshold model: first, both are probit threshold models, but the logistic model assumes a PDF that is the derivative of the sigmoid function, whereas the liability model assumes a standard normal PDF that is better supported by the central limit theorem, and second, previous studies have suggested negative correlations exist between entire PRSs and rare CNVs in schizophrenia and ADHD. However, we raise additional caveats, including that all cohorts may be slightly heterogeneous due to imperfect sampling, resulting in residual correlation differences between cases and controls. For these reasons we suggest that the scores taken from 0 and the expected value of CLiP serve as upper and lower bounds on the true heterogeneity of the cohort. As current uses of BUHMBOX assume 0 for all scenarios, it is likely still that these applications are underestimating the score.

• As low power is cited as a prior limitation to detect heterogeneity, an analysis evaluating power should also be included in the results section to weigh the merits of this new method.

As described in our response to point 3, we have included power calculations for the CLiP simulations in Figure 2 as well as p-values for all PGC cohorts evaluated in Table 1.

• In general, it would be helpful to expand upon the schizophrenia analysis to fully demonstrate the underlying factors of heterogeneity. This would provide more context for the usefulness of this method and what underlying issues or future directions it can uncover. A discussion of these aspects would make this manuscript more relevant for a general genetics audience and increase the likelihood of adoption.

With the available PGC data, we subdivided individual cohorts into ancestry groups using the ancestry inference tool fastStructure. For the four largest cohorts in the PGC data, we calculated the heterogeneity scores of the cases and controls likely to fall into each inferred subgroup and compared them against the score calculated over the entire cohort. The results of these tests are shown in Supplementary Figure S7. We did not see a significant decrease in heterogeneity when separating cohorts by ancestry, suggesting that ancestry is not the primary source of observed heterogeneity in these cohorts. We could not perform the same analysis for sex as that information was missing from one cohort (s234) and another cohort (clm2) comprised almost entirely males.

Minor comments:
• Page 11, lines 203-204 refer to Figure 3, but it is unclear which panels they refer to. Figure 3B and Figure 3C are not referenced directly in the text.

We have added specific labels for subfigures (now Figure 4), along with additional text to clarify the results.

• Page 8, line 150 has “heterogeneity” misspelled.

Corrected
• There are currently no methods detailing the studies in PGC. For example, were all participants of European ancestry and similar ages? Similar diagnostic criteria? This would help in the interpretation of results.

We have added a brief description of the cohorts in the Methods section on page 25 including ancestry and sex information, along with a reference to per-cohort details found in Ripke et al. (2014). Unfortunately, we could not consistently find details on age distributions for each cohort, and sex information was missing for one cohort (s234), but we have included age and sex as a possible source of confounding heterogeneity in the Discussion section.

• The word “cohort” is used for both the “cases” (comprised of true and false cases) and the overall sample size of “cases” and “controls” This can be confusing at times and alternative language should be used to distinguish between the samples.

We have removed all instances of “cohort” referencing only cases or controls and instead use it to refer to only the entire case/control data set. We now use “case set” or “cases” only to refer to affected individuals.

Reviewer #2: This study presents a method called CLiP which uses the correlations between SNPs (or predicted expression levels) in case patients to dissect heterogeneous subsets in cases. This is a generalization of Han et al method. The authors’ contributions are two: (1) they showed that the correlation=0 assumption under no heterogeneity is only valid in multiplicative model but not in liability threshold model and presented a correction for that. (2) they generalized the method to a) when other predicting variables than SNPs are used and b) when continuous phenotype is of interest.

Overall, the paper is quite well written. The problem is interesting and important, and the solution is straightforward. I find contribution (2) quite interesting. Contribution (1) is also important, but there can be a bit of controversy. First, the authors say that the correlation would be negative if “logistic or liability threshold model” is true. In liability threshold model, it is clear that the correlation becomes negative. For logistic model, I didn’t understand why the correlation has to be negative. In fact, Risch’s multiplicative model can be presented as logistic model. Multiplicative nature of ORs of SNPs can be modeled as additive in logit space. This seems to be written in the latter part of Supplementary Note section 9.2, but the long derivation was hard to follow. Probably the authors can give an intuitive explanation about that? Second, I personally agree that liability threshold model more closely models the truth, but am not sure if it’s really the truth. The truth might be in-between of the two (liability threshold model and multiplicative). I feel that the popularity of liability threshold model was driven from the heritability estimation (where liability is a must for binary traits). But that’s because liability is one reasonable explanation that can model heritability, but not because liability is strictly true. In sum, no one knows if that’s true. I am not sure if there were any studies that tried to test if liability threshold model more closely models the truth in complex diseases. It would be better if the authors can do some literature search and cite some. (If there were no such studies, I think extension of CLiP can be one method in the future.) I agree that Han et al. method can be underpowered by non-multiplicative model (e.g. liability), but worry if CLiP can increase false positive rate if the truth would be in-between.

We would like to greatly thank the reviewer for raising this issue, as it prompted us to reevaluate assumptions about the equivalence of the logistic and liability threshold models. As we describe in the text, these models have been shown previously to report similar odds ratios/effect sizes and disease risks for the same data set (Wray, 2010), and we believed since the logistic model itself can be interpreted as a thresholded model, with a sigmoid function instead of a normal CDF, that negative correlations would be observed at similar magnitudes as in the liability model. In the new Figure 1, we indeed observe that simulated data from a logistic model and its conversion to a liability threshold model produce similar levels of ascertainment of risk alleles. But while both models produce negative correlations between SNPs which increase with increasing odds ratio, the magnitude of the correlations is significantly larger in the liability model, whereas for the range expected of polygenic effects, those of the logistic model are sufficiently small to be assumed uncorrelated.

In light of these findings we have adopted the reviewer’s intuition that the expected value of CLiP and the expected value of 0 serve as lower and upper bounds on the true expected score of a homogeneous cohort. We elaborate on this point and additional caveats in a new section in the Discussion. We also include some points in favor of the liability model over the logistic model: namely, if both of these models are additive, the central limit theorem would suggest an underlying normal PDF is expected over the sigmoid derivative. Additionally, some evidence from studies comparing PRSs of cases with and without rare CNVs suggest that common alleles and rare CNVs are anti-correlated.

In the method, I expected some equations to derive the negative expected correlations under the liability model, but was surprised that they were skipped directly to CLiP-X, leaving relatively short contents for the main CLiP. I feel that this part is as important as CLiP-X and Y, so hope that a summary of 9.4 can be in the main method, so that readers can easily implement the CLiP method using their own code.
We agree that this section ends too abruptly, without addressing the calculation of expected values specifically in CLiP. We have expanded this section with a condensed version of the equations in supplementary section 9.4 but applying specifically to SNPs, so that expectations are expressed as sums over allele counts rather than integrals.

Minor comments:
The title is confusing. I didn’t catch what “polygenic risk score predictor” means. Given title, I expected contents related to the (currently popular) usage of PRS for disease predictions. I understand that the authors did not want to use SNP or locus to emphasize that expressions can be used, but not sure if the current title is the best choice.
Yes, our original intention was to indicate that the choice of variable was not restricted to SNPs, but we agree that the title may cause confusion. We have changed the title to “Leveraging correlations between variants in polygenic risk scores to detect heterogeneity in GWAS cohorts.”

The authors need to add more detailed and elaborated explanations about the “transcriptome-wide association study”. First, many geneticists might not be familiar with TWAS. The authors need to explain that there is an effort to associate the gene expression level of each gene with the phenotype. Second, to some people (like me), TWAS has double meaning: method name and study design name, so can be easily confusing.

Thank you for this suggestion. We have added a paragraph with a brief summary of TWAS and its relationship to CLiP-X on page 15, where CLiP-X is first encountered in detail. We agree that the added background information aids in the interpretation of the results, such as the meaning of the SNP-gene and gene-trait variance explained.

The method depends on the independency of SNPs. Did the authors do some LD pruning? Subtracting the control correlations might help controlling for long-range LD; was this the case in real data? Or controls are actually not needed?

We input only SNPs which have been reported in summary statistics, which presumably have already been LD pruned and from different loci. This is true of the 108 loci associated with schizophrenia used in our analysis. Subtracting the control score is intended to handle any lingering long-range LD, but it is also important to correct for other confounding sources of heterogeneity, such as batch effects or ancestry stratification within the same cohort. We have added a note about independence of SNPs in the Methods section of CLiP on page 27.

P9. Line 173: PRSs are expected to be zero than positive → you mean, negative?
Thank you: this was an error. We have switched “positive” → “negative”.

P11. Line 209: when the contents refer to Figure 3, I didn’t find it relevant. Is this a correct figure number?

The section was missing some labels for subfigures (now Figure 4). We have added these, as well as additional text to clarify each panel of the subfigure.

Reviewer #3: The manuscript considers a statistical method to detect phenotypic heterogeneity in GWAS cohorts by using individual-level genotype data on GWAS samples. The idea is that the correlation between genotypes at different SNPs in cases increases if the cases contain heterogeneous subgroups. The method is applied to schizophrenia data. The manuscript also extends the method to continuous traits but no real data examples are given.

I find it difficult to evaluate the novel contribution of this work because the method is not presented very clearly (see below for details) and it is unclear how big a difference to the existing method is in realistic settings. The continuous versions CLiP-X and CLiP-Y seem experimental and no real data example is given. The current real data example with schizophrenia GWAS does not convincingly show how the method could conclude whether there is or is not heterogeneity in cohorts.

We greatly appreciate the comments raised by the reviewer including comparison to the existing BUHMBOX implementation, lack of real data analysis in CLiP-X and CLiP-Y, and interpretation of results of the schizophrenia analysis. As described in our response to Reviewer 1, we recently obtained access to the UK Biobank, and we were able to apply heterogeneity detection in CLiP-Y to neuroticism as a quantitative trait. The results of this analysis are shown in Table 2. Unfortunately, we could not provide an analysis in real data for CLiP-X. We envision a primary application of CLiP-X to be TWAS, in which imputed gene expression variables for a large cohort are regressed against disease status. However, the fraction of disease variance explained for risk scores over gene expression imputed by TWAS is currently very low across many traits, and so CLiP-X is underpowered to detect heterogeneity in these data.

Regarding interpretability of heterogeneity detected by CLiP in schizophrenia, we agree that this does not allow a definitive conclusion that schizophrenia is etiologically heterogeneous, as the heterogeneity could be a result of non-disease related clustering that is not fully corrected by subtraction of controls. However, it does raise the question of possible underestimation of heterogeneity signals by BUHMBOX when assuming a null of 0 correlations between SNPs. We observe this in Table 1, where we have added p-values taken from a null score of 0 as a comparison to BUHMBOX: for all cohorts, these p-values do not pass the 0.05 threshold of significance, indicating the significance of model selection. We have added these caveats to the Discussion section.

About the term “controls”
Unclear whether “controls” in this paper refers to a sample from the population or to proper disease-free controls. If former, then should talk about “population controls” and make clear that these are not proper disease-free controls. Theoretically, this has an important effect on whether risk SNPs are expected to be independent in “controls”. Typically, SNPs at different loci are independent in general POPULATION that is a mixture of cases and disease-free controls. And when risk SNPs are independent in population they are not independent either in cases nor in disease-free controls.
Thank you for raising this clarification. We use population controls with no filtering of individuals based on disease status for all simulations, and we have added this clarification to the main text where appropriate to reflect this. SNPs in these controls are generated completely independently, so the expected correlation is 0. Additionally, in all simulations except for those in Figure 1A (now Supplementary Figure S2A) we use a relatively low prevalence of 0.01 corresponding to that observed of schizophrenia. Given the low disease prevalence, we would not expect a significant difference in correlation between population controls and disease-free controls.

Models:
Three disease models are mentioned: “multiplicative Risch/binary model”, “logistic model” and “liability threshold model”.

The manuscript does not define clearly “multiplicative (Risch/binary) model”, or rather what is its difference from the logistic model. This should be done in Methods.
Best proxy is line 131 saying: “a multiplicative binary model, in which an individual's risk is the product of odds ratios of probability of disease for associated SNPs”
Thank you for this suggestion. We have included a section at the start of the Methods explicitly defining the Risch, logistic, and liability threshold models.

First, risk is a product of relative risks, not odds-ratios. I assume that SNP effects are defined in terms of ORs and not RRs and so the multiplicative model should be formulated for odds of disease?
This is correct: the Risch model does involve a product of relative risks rather than odds ratios. We have added to the Results section a calculation of an approximate relative risk given an odds ratio and the disease prevalence described in Zhang and Kai (1998). With a prevalence of 0.05, this approximation converts an odds ratio of 1.16 to a relative risk of 1.15. With a prevalence of 0.05 and odds ratio of 1.06, the relative risk is 1.059. Additionally, it has been previously reported that ORs can be substituted for RRs if the prevalence is small (Wang, 2013). For these reasons we found it appropriate to substitute the odds ratios for relative risks in our simulation. We have added additional details about this nuance to the main text on page 26.

Second, there needs to be some baseline risk for an individual without any risk allele on which each risk allele adds to. That together with relative risks or odds-ratios and genotype distribution in population define the prevalence in the population. Unclear what is the population prevalence in the example of Figure 1A and whether prevalence of disease is matched between the “multiplicative model” and “logistic model”. Both prevalence and ORs used in simulations should be realistic.

In the original Figure 1A (now Supplementary Figure S2A), the population prevalence for both logistic and Risch models were set to 0.05. The prevalence is the constant term in the Risch model and can be set directly. In the logistic model, we adjusted the intercept term such that without observing any genotype data, the probability of being a case is set to the population prevalence. An explanation of this has been added to the Methods section on page 26.

We had initially intended for this figure to demonstrate the difference between logistic and Risch models, so we used parameters that maximized this difference. The value of 1.16 was chosen in particular so that a hypothetical individual with the maximum number of risk alleles and thus the maximum possible disease risk, given by (0.05)*(1.16)^(2*10) = 0.97, is smaller than 1. However, in light the difference in the expected correlations when comparing logistic and liability models, with the logistic model producing correlations near 0, we reran this simulation with more realistic parameters: a prevalence of 0.01, effect allele frequency of 0.2, and odds ratio of 1.06, the average value of the 108 loci reported by the PGC for schizophrenia. This result alongside the original large OR test is now shown in Supplementary Figure S2B. With more realistic parameters, the correlation bias among homogeneous cases is indeed near 0 when simulating from a logistic model.

Third, the logistic regression says that odds of disease increase multiplicatively with each copy of the risk allele, and multiplication happens independently across the SNPs. Thus I see no difference between “multiplicative model” and “logistic regression model” as statistical models. I assume that the difference is that the assumption about the population genotype distribution are different in settings where authors use these two models. In settings with “multiplicative model” the authors assume that risk SNPs are independent in CASES (theoretically an unrealistic assumption), whereas in settings with “logistic model” the assumption is that SNPs are independent in the whole POPULATION (realistic assumption). However, when prevalence is low (a few percent or less) then independence between SNPs in population together with the logistic regression model lead to approximate independence of SNPs in CASES (as well as in disease-free CONTROLS), see e.g. Pirinen et al. Nat Gen 44, 848–851(2012). Thus, if prevalence is realistic for typical common diseases, I would not expect a large difference from independence in cases, and hence no large negative correlations between SNPs in cases. The paper should make clear whether the theoretically correct fact of negative risk SNP correlations in cases truly has a practical effect over earlier method (BUHMBOX) when prevalence is realistic. The simulations in Figure 1A should be done so that both settings (“multiplicative” and “logistic”) have the same population prevalence and ORs, and then it should be reported whether there is a practical difference after the correction for negative correlations in cases.

As we described in response to reviewer 2, this concern prompted us to further investigate the expected correlations of SNPs in the logistic model, which we detail in a new section of the Results on page 7. Our new Figure 1 shows that while negative correlations within cases are still expected of logistic models, for small effect sizes these biases are small enough that they can be ignored, which is in line with the conclusion of Pirnen et al. In the liability threshold model, however, significant negative correlations are expected even with a low prevalence (0.01 in Figure 1). We have amended the manuscript to state that CLiP applies specifically to assumptions of the liability threshold model, and CLiP and BUHMBOX together serve as a lower and upper bound on the true expected score of a homogeneous cohort.

The simulations in Figure 1A have been moved to the supplement and is now Figure S2. We have included another test with parameters more realistic of polygenic traits: prevalence of 0.01, odds ratio of 1.06, and allele frequency of 0.2; these values produce nearly no negative correlations, as expected of our new Figure 1 and Pirnen et al.

Fig.2 “Colors indicate the total cohort size. All tests were conducted over 50,000 cases and 50,000 controls”
Inconsistent that sample varies and is fixed to 50k.

The reference to 50k simulated cases and controls was a mistake: this applies to Figure 2B but not 2A. The sentence now reads “All tests were conducted with a SNP variance explained of 0.05.”

Methods section mentions one score: S_het, “modified heterogeneity score”. Is this what is used in all Figures? Then should use symbol “S_het” in main text to make this clear.
In the methods section of CLIP, we have replaced S_{het} with S_{CLIP}. To match this to Figure 2, S_{CLIP} is the difference between the green and blue lines. Where the score is referenced in the CLIP-Y section, it is now represented as S_{CLIP-Y}.

L.136 “conventional, case-control score for heterogeneity”
This is not defined anywhere in Methods.
We intended for this phrase to refer to the original Buhmbox method proposed in Han et al. 2016. This sentence now reads, “We first tested the unmodified Buhmbox method, as implemented in Han et al. [30], with cases generated from a full logistic model as well as the multiplicative Risch model.”

L. 138 “We simulated individual data using 100 independent SNPs”
Independent in population, or in cases or in controls?
The SNPs are independent in the population, and not necessarily independent in cases and controls. This sentence now reads, “We simulated individual data by sampling 100 independent-by-population SNPs whose effects were standard-normally distributed.”

L.140 “we assumed a variance explained of 1”
Needs explanation. I assume that this means that the phenotype is deterministically defined from genotypes. ORs are now the same but do both models also lead to the same prevalence in the population?

We had intended to convey that the simulation procedure for the logistic and Risch models differs from that of the liability threshold model because a normally distributed error term is not explicitly sampled and added to the log odds for each individual. But since this is standard practice for these models anyway, we think this sentence instead only adds confusion, and so we have deleted it.

The question of prevalence between the logistic and Risch models is an interesting one. For any given log odds x_i, the two models express the disease risk differently [sigmoid(x_i) vs exp(x_i)]. To make the two models comparable, we set parameters so that the disease risk prior to observing any genotypes is equal to a fixed prevalence of 0.05. In the Risch model, the prevalence term can be set directly. In the logistic model, the intercept term $\beta_{0}$can be set such that

$P\left( y_{i}=1 \right)=\frac{1}{\left( 1+exp\left( -\beta_{0} \right) \right)}\Rightarrow\beta_{0}=-log\left( \frac{1}{P\left( y_{i}=1 \right)}-1 \right)$

We have added a paragraph addressing this in the main text on page 26.

L. 141 “We evaluated the dichotomous-trait score relative to sample sizes for both homogeneous and heterogeneous groups.”
Unclear what this means?

This sentence has been replaced with “We evaluated the existing Buhmbox method in simulated homogeneous and heterogeneous cohorts. Heterogeneous cases comprise a mixture of true cases and controls generated from either the logistic or Risch models.”

“case cohorts were generated by repeatedly sampling control genotypes and selecting individuals whose PRS pass a threshold corresponding to a prevalence of 0.01”
I assume that genotypes are sampled from POPULATION frequencies, not from CONTROL frequencies? Or if controls are population samples i.e. a mixture of cases and proper controls, then that should be said very clearly.

Yes, these genotypes were sampled from population frequencies. We have added the sentence, “SNPs of control cohorts were sampled independently according to population allele frequencies.” prior to this section.

L.153 “PRS over 108 genomewide-signicant SNPs with a total variance explained of 0.034”
On which scale is this variance? And is it in population or in some case-control sample?
The variance is scaled to a standard normal hidden liability distribution with total variance of 1. The magnitudes of our simulated SNP effects are scaled such that the variance of the PRS exactly equals 0.034, and we then add a normally distributed error of 0.966 to obtain the liability distribution. This point is addressed in an added section introducing the liability threshold model on page 26. The mean and variance of this liability distribution rely only on population allele frequencies of independent SNPs and have no dependence on any case-control sample.

L.185 CliP-Y section starts: “In practice, we found converting PRSs to percentiles improved performance for all learned weight functions, possibly because percentiles limit the domain of the PRS function over which the function must be 0, and they reduce the contribution to the score calculation by extreme PRS values.”

Some kind of intro to what this section aims to do and what weight functions etc are should be stated before the sentence above, that currently is incomprehensible when first read by the reader who has not yet read the Methods that come only later.
Thank you for this suggestion - we had originally written this section assuming it was preceded by the Methods. We have added a brief paragraph giving an overview of the task addressed by CLiP-Y.

L 293. “the presence of distinct mixtures of cases within a cohort which have been identified as cases through different PRSs.”
Unclear whether this means that the cases are identified through different PRSs or whether these cases happen to have heterogeneity and hence different distributions of PRSs?

We imagine the cases have been generated according to different PRSs corresponding to their distinct subtypes, but these PRSs are unknown during discovery. We have replaced this sentence with the following: “In a case-control GWAS, a heterogeneous cohort of cases can be interpreted as comprising a mixture of hidden case subtypes, each exhibiting an elevated risks of disease according to a unique polygenic risk score. These subtype-specific PRSs are unobserved and may confound discovery of case-control associations.”

“misclassifcation, whereby a subset of individuals are not really cases, but have been rather labeled as such despite genetically being controls”
Why there is word “genetically” there? Missclassification happens purely by mislabeling the phenotype, and is not informed by genotypes.
We have removed the word “genetically” from this sentence.

L 296. “but it may also suggest distinct disease etiologies, some of which are not ascertained for the PRS of interest. Second, a mixture of unobserved sub-phenotypes with distinct PRSs.”
What is the difference here? Both refer to a setting where sub-phenotypes of cases have different effect sizes (“distinct PRSs”).

This distinction is important primarily due to the resulting correlation patterns produced between SNPs of cases, which affects the ability to detect underlying heterogeneity using CLiP. While both scenarios refer to subgroups having distinct PRSs, the first scenario involves at least one subgroup appearing as population controls in relation to the disease over the set of associated SNPs of interest. This may occur if there exist multiple subtypes of a disease with distinct risk factors, and not all of those subtypes are associated with a given PRS. For example, a case may acquire a disease through non-genetic means or simply by a different genetic etiology than the one captured by the PRS. The second scenario involves multiple subgroups each appearing as cases, but each subgroup has a significant number of unique SNP associations. Correlations between all pairs of SNPs will be positive in scenario 1, because all SNPs are ascertained over the same individuals, but correlations between two SNPs associated with two different subgroup PRSs will be negatively correlated, attenuating the score when all correlations are summed. Heterogeneity in both scenarios will skew the CLIP score towards less negative values than that expected of homogeneous cases, but less so in scenario 2 than in scenario 1.

We have modified the second sentence to make this distinction clearer. It now reads, “Second, a mixture of multiple unobserved sub-phenotypes which are all captured to some degree by the PRS of interest. An idealized example would involve SNPs of a PRS divided between sub-types such that each SNP was truly associated with only one subtype, and no SNP shared associations with multiple subtypes.” We hope this makes explicit the distinction from the first scenario, in which one sub-type appears as population controls with respect to the PRS.

L 326. “The expected correlation among controls, E [R0jk], is always 0 in practice as SNPs are sampled independently, but is included below for clarity.”
If you assume that SNPs are independent in POPULATION, and that the logistic model holds, then risk SNPs are independent neither in disease-free CONTROLS nor in CASES. (But if prevalence is low, they are approximately independent in both disease-free CONTROLS and in CASES. And if you mean by controls the population sample then the sentence is true, but uses unclear terms.)
Yes, we intend controls to refer to population controls, whose SNPs are sampled independently according to population allele frequencies and which include a fraction of true cases in accordance with disease prevalence. So the expected correlation should be 0. We also use a prevalence of 0.01 in all simulations, so in practice the difference in correlations between true controls and population controls is minor.

We have replaced this sentence with the following: “If we interpret controls as population controls in that they are sampled from the full liability distribution, then the expected correlation among controls E[R0jk] is always 0 as SNPs are sampled independently.”


Supplementary material:


L.759-760 For which genotype group Is prevalence B given? Odds-ratios do not directly correspond to risk, but to odds.
(27) and elsewhere would be clearer is conditioning on being case was explicitly written out.

(28) This equation is based on the ASSUMPTION that p(x_i,x_j) = p(x_i)*p(x_j) in cases. If this is the assumption of "Risch model", then that should be simply stated, and there is no need to show mathematically that the SNPs are independent if that is already assumed.

We agree that the section about the Risch model is redundant and have removed it to avoid confusion. The prevalence B is not used in the rest of the proof.

(33) Assumes Hardy-Weinberg equilibrium in cases. It is not self-evident that this is true, so it should be made clear that this is an assumption.

Thank you: we have added that Hardy-Weinberg equilibrium is assumed in cases here as well as in section 9.4, where binomial-distributed SNPs are also assumed.

Formulas 776-778 should be corrected for “=0” parts.

Thank you, this has been corrected.

(35) Would benefit from middle steps that say that this is Bayes rule.

We have added two intermediate steps and a mention of Bayes rule in this part of the proof.

Formulas (51) – (54) are not understandable as there are unexplained indexes (s and sc) and also likely something is missing.
(55) conditions on X_i = b, and right hand side has alpha_i and X_i and T (which is not defined anywhere).

I feel there is too much work left for reviewer trying to understand unclear and undefined notation and formulas. I haven’t read supplementary further.

We would like to thank the reviewer for catching these mistakes in the notation: it appears some parts were omitted when transferring this section between the Methods and Supplement. We have added the missing information and provided a more detailed explanation of the motivation and variables.

**Have all data underlying the figures and results presented in the manuscript been provided?**
Large-scale datasets should be made available via a public repository as described in the *PLOS Genetics* [data availability policy](http://journals.plos.org/plosgenetics/s/data-availability), and numerical data that underlies graphs or summary statistics should be provided in spreadsheet form as supporting information.

Reviewer #1: Yes

Reviewer #2: Yes

Reviewer #3: Yes

PLOS authors have the option to publish the peer review history of their article ([what does this mean?](https://journals.plos.org/plosgenetics/s/editorial-and-peer-review-process#_blank)). If published, this will include your full peer review and any attached files.


If you choose “no”, your identity will remain anonymous but your review may still be made public.


**Do you want your identity to be public for this peer review?** For information about this choice, including consent withdrawal, please see our [Privacy Policy](https://www.plos.org/privacy-policy).

Reviewer #1: No

Reviewer #2: No

Reviewer #3: No
