# Supplementary material for: Leveraging correlations between variants in polygenic risk scores to detect heterogeneity in GWAS cohorts
Source: PLoS Genet. 2020 Sep 21;16(9):e1009015. doi: 10.1371/journal.pgen.1009015 (PMC7529195; doi:10.1371/journal.pgen.1009015)
Supplement: Attachment — Submitted filename: PLOS_genetics_heterogeneity_comments_round2.docx [file pgen.1009015.s023.docx]

Reviewer #2: The authors have addressed my comments.
The current version looks much better, particularly in describing the models (logistic / liability thres)

Reviewer #3: I thank the Authors for expanding the manuscript and clarified the methods considerably. The manuscript has interesting methodological ideas that are useful for the field.

Main comments:

1. Such a clearly differing behavior of correlation between predictors under logistic and probit models seems surprising. I would still suggest that the Authors check that they are simulating data from the same populations under both models.
In particular, on p.26 l.546-547 it sounds like the prevalence in logistic model has been set for the rare set of individuals who carry no risk alleles at all, and hence the population prevalence will become much larger than the prevalence in those genetically protected extreme individuals. (Unless genotypes have been mean-centered in logistic model as they seem to be in liability threshold model formula 3.) The same issue with Risch model where Authors say in their answers that “maximum possible risk is given by (0.05)*(1.16)^(2*10) = 0.97”, but with this approach the prevalence in the population would rather be
23% = sum( dbinom(0:20, size = 20, prob = 0.5) * 0.05 * 1.16^ (0:20) ) than target 5%.
Similarly you could compute the population prevalence that the logistic regression model is imposing as the expectation of probability of being case (formula 2) over the population genotype distribution.

If there are differences between population prevalence in logistic and liability threshold model simulation in Figure 1, that could affect the observed differences in SNP correlations in cases. My suggestion is that Authors check these simulations once more to make sure that both models are simulating genotype data from a population where the POPULATION PREVALENCE is 5% (or 1%) and the effect sizes are the same per allele.

(Note: All page and line numbers refer to the marked-up versions of the manuscript and supplement. Changes are highlighted in blue.)

Thank you for this detailed observation. We do mean-center the genotypes in logistic regression, but we neglected to specify this in equation 2 as we did in the liability threshold model in equation 3. In equation 2 (p. 25, line 546) we have replaced $X_{\left( m \right)}$ with $\left( X_{\left( m \right)}-E\left[ X_{m} \right] \right)$.

We also wanted to confirm that the resulting prevalences when sampling from both models are indeed the same. To this end we have added a new Supplemental Figure 2 on page 63 which shows single randomly sampled cohorts with case/control labels assigned by each model and the resulting percentage of cases. There is a slight inflation toward higher prevalences in logistic regression at higher ORs (max 0.0175 vs an intended prevalence of 0.01 at OR=1.6), but the inflation is small, and it appears to coincide with the slight attenuation of ORs in the logistic model in Figure 1A. As we describe in the preceding paragraph starting on page 61, line 1145 in the Supplement, we think this happens because the logistic model does not account for the variance of the log-odds. Large odds ratios will produce a wider log-odds distribution, so a larger percentage of samples are assigned to cases. This inflation in prevalence does not appear to be primarily responsible for the significant difference in correlation between logistic and liability threshold models because the correlations begin to differ even at low odds ratios where the inflation negligible.

Additionally, the Reviewer is correct that we did not apply the same correction to simulations from the Risch model, so the prevalence of simulated cohorts is indeed inflated. We have amended the methods section for the Risch model (p. 25, line 530 in the main text) so that the constant A is not equal to the prevalence, but rather the prevalence divided by an approximation of the expected value of the contribution to the odds by all SNPs. We have added a derivation of this expected value in the Supplemental Information starting on page 63, line 1165 (Section 8.7). We also moved our analysis of relative risk vs odd ratios from the Methods to this section on page 64, which is why this paragraph is crossed out in Section 4.1 of the main text (p. 27). We also re-ran the Risch simulations in Supplemental Figure 2 (now 3) on page 65, now with the correct prevalence estimate, and we do not see a significant difference in the resulting heterogeneity scores.

Also, applying the aforementioned adjustment to the Risch model frees us from constraining the number and magnitude of SNP odds ratios so that the max value is less than 1. Therefore, in both the Risch and logistic models in Supplemental Figure 2A (now 3A) on page 65, we rounded the fixed odds ratio from 1.16 to 1.2. To make panel A more comparable with panel B, we changed the number of SNPs from 10 to 100, changed the fixed effect allele frequency from 0.5 to 0.2, and changed the prevalence to 0.01, so that the only difference between the simulations in the two panels is now only the odds ratios (1.06 vs 1.2).

2. Terminology:
Usually “probit model” means a liability threshold model with Gaussian CDF, and this model is often called simply “liability threshold model” as is done also in this manuscript. Logistic regression model is also a version of liability threshold model, although not typically called as such, but it seems confusing to me if logistic regression is now called a “probit model” as on p.7 l.151-152 and p.27 l. 564.
The end of sentence on l.152 “dot product … sigma(1-sigma)” is unclear.

The Reviewer is correct that the probit model exclusively refers to a Gaussian CDF, so we cannot use that term to draw a similarity between the two models. We have modified both sections on page 7 and page 27 so that these models are now described as generalized linear models with S-shaped link functions. We think this is a more technically accurate description which also conveys that the logistic and liability threshold models are conceptually very similar. The sentence on line 152 referring to “sigma(1-sigma)” has also been removed.

3. Figure 1:
Are the colors wrong way around in the figure? Or are the results wrong way around?
In panel B, blue has larger negative bias but the legend says blue is logistic model and that in B logistic model is having "notably smaller negative bias"?
Similarly in C, blue seems Normal and cyan seems logistic?

Thank you for catching this. The legend (page 9) is indeed incorrect: blue should denote the liability threshold model and cyan the logistic regression model. The labeling in C is correct with blue indicating normal (liability) and cyan indicating sigmoid derivative (logistic). We have added a phrase in B stating the same. Adding to the confusion is our ambiguous use of the descriptor “smaller” in “the negative bias is notably smaller in the logistic model,” which we now understand can be interpreted the opposite way, that the score itself is more negative. We have changed this phrase so that the score of the logistic model is described as “less negative.”

4. l.754-755. Where is it shown that CliP score ~ N(0,1) under the null. Section 4.2. does not mention that at all.

We have added a brief description at the end of Section 4.2 on page 29 addressing normality of the CLiP score. We refer to discussion in Han et al. (2016) showing the BUHMBOX score is standard normally distributed, and as CLiP modifies the score by subtracting a constant term representing the correct expected null score, it should be distributed with the same variance.

We also noticed that the statement in line 754-755 (now line 783-785) is itself inaccurate if the correction to the mean score in CLiP is applied, as the distribution of the score under the null would not be standard normal because it does not have a mean of 0. We have modified this sentence to address this point.

Minor comments:

p.28 “The case probabilities conditioned on SNP values P (y=1|X j ) are calculated from the liability threshold model in equation 3.”
How do you get the effect sizes on liability scale? Refer to the equation that you use later in formula 29.

Thank you, we have added a reference to equation 29 in this paragraph.

Supp. Text
L.993 “{1}^N” misses “0”

Corrected

L.995 One assumption not mentioned here is that genotypes at different loci are uncorrelated in the population.

We have added an additional sentence in this paragraph (now p. 50, line 1013) stating that genotypes are uncorrelated in controls.

We also made a correction on page 8, line 160 in the Results: the simulation parameters in Figure 1 should be 10 SNPs and allele frequency 0.5, not 100 SNPs and allele frequency 0.2. The latter parameters apply to Figure 2, which used to be Figure 1 in our initial submission.
